# Characterization of the SigD Regulon of *C. difficile* and Its Positive Control of Toxin Production through the Regulation of *tcdR*

**DOI:** 10.1371/journal.pone.0083748

**Published:** 2013-12-16

**Authors:** Imane El Meouche, Johann Peltier, Marc Monot, Olga Soutourina, Martine Pestel-Caron, Bruno Dupuy, Jean-Louis Pons

**Affiliations:** 1 Laboratoire G.R.A.M. (EA 2656 IFR 23 IHURBM), Université de Rouen, Rouen, France; 2 Laboratoire Pathogenèse des Bactéries Anaérobies, Institut Pasteur, Paris, France; 3 Université Paris Diderot, Sorbonne Paris Cité, Cellule Pasteur, Paris, France; 4 Laboratoire Ecosystème intestinal, Probiotiques, Antibiotiques (EA 4065, IFR IMTCE), Université Paris Descartes, Paris, France; Robert Koch Institut, Germany

## Abstract

*Clostridium difficile* intestinal disease is mediated largely by the actions of toxins A (TcdA) and B (TcdB), whose production occurs after the initial steps of colonization involving different surface or flagellar proteins. In *B. subtilis*, the sigma factor SigD controls flagellar synthesis, motility, and vegetative autolysins. A homolog of SigD encoding gene is present in the *C.difficile* 630 genome. We constructed a *sigD* mutant in *C. difficile* 630 ∆*erm* to analyze the regulon of SigD using a global transcriptomic approach. A total of 103 genes were differentially expressed between the wild-type and the *sigD* mutant, including genes involved in motility, metabolism and regulation. In addition, the *sigD* mutant displayed decreased expression of genes involved in flagellar biosynthesis, and also of genes encoding TcdA and TcdB as well as TcdR, the positive regulator of the toxins. Genomic analysis and RACE-PCR experiments allowed us to characterize promoter sequences of direct target genes of SigD including *tcdR* and to identify the SigD consensus. We then established that SigD positively regulates toxin expression via direct control of *tcdR* transcription. Interestingly, the overexpression of FlgM, a putative anti-SigD factor, inhibited the positive regulation of motility and toxin synthesis by SigD. Thus, SigD appears to be the first positive regulator of the toxin synthesis in *C. difficile*.

## Introduction

*Clostridium difficile* is a Gram positive, anaerobic, spore-forming bacterium recognized as the major etiological agent of intestinal diseases associated with antibiotic therapy, with clinical manifestations ranging from diarrhea to pseudomembranous colitis [1]. The disruption of the commensal intestinal flora by antimicrobial therapy allows colonization of the intestinal tract by *C. difficile* [2]. Spores germinate, vegetative cells multiply and toxigenic strains produce two toxins, TcdA and TcdB, considered as major virulence factors, which are responsible for intestinal damage [3]. The epidemiology and severity of *C. difficile* infections has evolved over the past ten years, mainly due to the emergence and spread of a so-called hypervirulent strain belonging to PCR-ribotype 027 [4].

The mechanisms of regulation of genes encoding virulence factors are of major interest in *C. difficile*, since the spectrum of intestinal disease is highly variable. Beyond intestinal colonization, toxin synthesis is the critical event in *C. difficile* intestinal disease. The toxin encoding genes *tcdA* and *tcdB* are located in a 19.6 kb pathogenicity locus [5], with three accessory genes encoding TcdR, TcdC and TcdE. TcdR is an alternative sigma factor that directs transcription from the *tcdA* and *tcdB* promoters [6]. TcdC is an anti-sigma factor that negatively regulates TcdR-dependent transcription [7], although its role in toxin synthesis is still controversial [8,9]. TcdE is a holin-like protein required in the release of the toxins from the cells [10], although its role has also been discussed [11]. Several global regulators, such as CcpA, CodY, Spo0A and SigH regulate expression of toxin genes in response to diverse environmental stimuli. CcpA represses toxin expression in response to PTS sugar availability by binding to the regulatory regions of the *tcdA* and *tcdB* genes [12], as well as regulatory regions of *tcdR* and *tcdC* genes [13]. CodY, which controls in *B. subtilis* many genes induced when cells make the transition from rapid exponential growth to stationary phase or sporulation, represses toxin gene expression by binding to the putative promoter region of the *tcdR* gene [14,15]. The role of Spo0A, the response regulator of sporulation initiation, in toxin production is still controversial [16,17]. Finally, the alternative sigma factor SigH, a key element in the control of the transition phase and of the initiation of sporulation, negatively modulates toxin and motility expression [18]. Most of these regulators control toxin genes expression in association with genes encoding major cell functions, suggesting a strong relationship between the physiology of *C. difficile* and the expression of the virulence factors of this bacterium.

Recently, Aubry et al. showed that regulation of the flagellar regulon differentially modulated toxin expression in *C. difficile* [19], according to a yet uncharacterized mechanism. The flagellar regulon of *C. difficile* includes a first region encoding late stage flagellar proteins such as FliC (filament protein) and FliD (capping protein), a second region containing flagellar glycan biosynthetic genes and a third region encoding the hook basal body proteins and resembling the *fla*/*che* operon of *B. subtilis* [20,21] (Figure S1). In *B. subtilis*, the expression of genes of the *fla/che* operon depends on a promoter P_A_ recognized by SigA and a promoter P_D-3_ recognized by SigD [22]. Besides regulation of motility genes in *B. subtilis*, SigD plays also an important role in the control of peptidoglycan-remodeling autolysins (LytC, LytD and LytF) [23].

The *C. difficile* 630 genome carries a gene (*CD0266*) encoding a putative SigD factor homologous to SigD of *B. subtilis*. In the present study, we first analyzed the gene expression profile of *C. difficile* wild-type compared to the *sigD* mutant and identified the consensus sequence of the SigD-controlled promoters. Then, we demonstrate the role of SigD as a direct and positive regulator of *tcdR* expression and consequently of toxin synthesis in *C. difficile*. Thus, we identified a SigD dependent consensus sequence upstream of *tcdR* gene and we showed that SigD positively acts on the *tcdR* transcription as an alternative sigma factor of the RNA polymerase. In support of this result we showed that the putative anti-SigD factor FlgM represses motility and toxin genes expression via the inhibition of SigD activity.

## Materials and Methods

### Bacterial strains and growth conditions

Bacterial strains and plasmids used in this study are presented in Table 1. *C. difficile* strains were cultured on blood agar (Oxoid), BHI agar (Difco), BHI broth (Difco) and TY mediumin an anaerobic environment (H_2_10%, CO_2_ 10%, N_2_80%) at 37°C. When necessary, cycloserine (250µg/ml), thiamphenicol (15µg/ml), erythromycin (5µg/ml) and anhydrotetracycline (ATc) (20ng/ml) were added to *C.difficile* cultures. *Escherichia coli* strains were cultured aerobically at 37°C in LB broth or LB agar (MP Biomedicals) containing chloramphenicol (25µg/ml) or ampicillin (100µg/ml) when required.

**Table 1:** Strains and plasmids used in this study.

**Strains/plasmids**	**Relevant features**	**Reference or source**
***C. difficile***		
630	wild type Erm**^*R*^**	[65]
630Δ*erm*	*C. difficile* 630, Erm**^*S*^**	[66]
630Δ*erm sigD::intron-erm*	Erm**^*R*^**	This study
630Δ*erm* + pMTL::P*CD2767*-*flgM*	Tm**^*R*^**	This study
630Δ*erm* + pMTL007	Tm**^*R*^**	This study
630 Δ*erm* + pMTL84121	Tm**^*R*^**	This study
*sigD* mutant + pMTL84121	Tm**^*R*^**	This study
630Δ*erm* + pRPF185	Tm**^*R*^** ATc**^*R*^**	This study
*sigD*::*erm* + pRPF185	Tm**^*R*^** ATc**^*R*^**	This study
*sigD*::*erm* + pRPF*-sigD*	Tm**^*R*^** ATc**^*R*^**	This study
*sigD::erm* + pRPF-*sigD to CD0272*	Tm**^*R*^** ATc**^*R*^**	This study
*sigD* mutant + pDIA5941	Tm**^*R*^**	This study
630*∆erm* + pDIA5941	Tm**^*R*^**	This study
***E. coli***		
TOP10	F^-^ *mcr*A D(*mrr-hsd*RMS-*mcr*BC) f80*lac*ZDM15 D*lac*X74 *deo*R recA1 *ara*D139 D(*ara-leu*)7697 *gal*K *rps*L(StrR) endA1 *nup*G	Invitrogen
HB101 (RP4)	supE44 *aa*14 *galK*2 *lacY*1 D(*gpt-proA*) 62 *rpsL*20 (Str**^*R*^**) *xyl-5 mtl-1 recA*13 D(*mcrC-mrr*) *hsdS*_B_(r_B_^-^m_B_^-^) RP4	Laboratory stock
M15	*E.coli* K-12 derivative containing plasmid pREP4.Providing a high levelof expressionof the *lac* repressor; Kan**^*r*^**	Qiagen
**Plasmids**		
RP4	Tra**^*+*^** IncP Ap^R^ Km^R^ Tc^R^	[67]
pMTL007	group II intron, ErmBtdRAM2 and ltrA ORF from pMTL20lacZTTErmBtdRAM2 Cm^R^	[24]
pMTL007::*sigD*-228s	Tm**^*R*^**	This study
pQE30	expression vector with hexa-His on N-terminal ; Ap^r^	Qiagen
pMTL84121	Tm**^*R*^**	[31]
pDIA5941	pMTL84121 derivative carrying *tcdR* with its promoter region	This study
pRPF185	Tm**^*R*^** ATc**^*R*^**	[25]
pRPF-*sigD*	pRPF185 derivative carrying *sigD* gene	This study
pRPF-*sigD* to *CD0272*	pRPF185 derivative carrying the*sigD*to *CD0272*genes	This study
pMTL::P*CD2767*-*flgM*	pMTL007 derivative containing *flgM* gene with PCD2767promoter	This study

### General DNA techniques

Chromosomal DNA extraction from *C. difficile* colonies was performed using the InstaGene Matrix kit (Bio-Rad). PCRs were carried out in a reaction volume of 25 µl using GoTaq Green Master (Promega) or FastStart High Fidelity PCR System (Roche). The primers used (Eurofins MWG Operon, Eurogentec) are listed in Table S1. PCR products and plasmids were purified using a NucleoSpin Extract II kit and a Nucleospin plasmid kit (Macherey-Nagel), respectively.

### RNA isolation and quantitative real time PCR

Total RNA of *C. difficile* was extracted with the RNeasy Mini kit (Qiagen). Samples were treated with two different DNases, DNase I (Sigma) and Turbo DNA-free kit (Ambion) according to the respective manufacturer’s instructions. The total RNA quantity and purity were spectrophotometrically measured (Nanovue, GEHealthcare) and two micrograms of total RNA was reverse transcribed using the Omniscript enzyme (Qiagen) and random 15-mer primers (Eurofins MWG Operon). A total of six nanograms of cDNA were used for subsequent PCR amplification with the IQ SYBR green Supermix (Bio-Rad) and the appropriate primers (0.5 µM final concentration). Specific primers used for PCR amplification were designed with Beacon Designer software (PREMIER Biosoft International) (Table S1). Quantification of 16S rRNA was used as an internal control. Amplification, detection (with automatic calculation of the threshold value), and real-time analysis were performed in duplicate and with three different RNA samples for each condition, by using the CFX96 real time PCR detection system (Bio-Rad). The value used for the comparison of gene expression levels was the number of PCR cycles required to reach the threshold cycle (*C*_*T*_). Expression of an mRNA species was calculated as fold changes using the formula: Fold changes = 2^-ΔΔCt^; with –ΔΔCt = (Ct _gene X_ – Ct _16S rRNA_) _mutant_ – (Ct _gene X_ – Ct _16S rRNA_) _wild-type_. Statistical analysis was performed with Student’s *t* test and a P value of ≤ 0.05 was considered significant.

### Construction of a *C. difficile sigD* mutant

The ClosTron system was used as described previously [24] to inactivate the *sigD* gene. Briefly, primers were designed (http://www.sigmaaldrich.com) to retarget the group II intron of pMTL007 to *sigD* (Table S2), and used to generate a 353 pb DNA fragment by overlap PCR according to the manufacturer’s instructions. These PCR products were cloned into the HindIII and BsrGI restriction sites of pMTL007 and sequenced to verify plasmid constructions with primers pMTL007seqF and pMTL007seqR. pMTL007::Cdi-*sigD*-228s was transformed into the conjugative *E. coli* HB101 (RP4) and then transferred via conjugation into *C. difficile* 630Δ*erm*. *C. difficile* transconjugants were selected by subculturing on BHI agar containing cycloserine and thiamphenicol. Then, the integration of the group II intron RNA into the *sigD* gene was induced and selected by plating onto BHI agar containing erythromycin. PCR using the primers ErmRAM-F and ErmRAM-R confirmed the erythromycin resistant phenotype due to the splicing of the group I intron from the group II intron following integration. To verify the insertion of group II intron in the *sigD* gene, we performed PCRs using (i) two primers flanking *sigD* (*sigD*-F-*sigD*-R), (ii) a primer in *sigD*, *sigD*-F and the intron primer EBSu and (iii) ErmRAM-F and ErmRAM-R (Table S1, Figure S2).

### Southern hybridization

For Southern blot analysis, 5 µg of genomic DNA from *C. difficile* strain 630∆erm and the *sigD* mutant strain were digested to completion with *Hin*dIII, subjected to agarose gel electrophoresis (0.8%) and then transferred from the gel onto Hybond-N+ filter (Amersham).The Southern blot probe was generated by PCR using pMTL007 plasmid as a template and primer pair OBD522 and OBD523 (Table S1), yielding a 374 bp PCR product that hybridizes within the group II intron. Southern blot analyses were performed using Amersham ECL Direct Nucleic Acid labeling and detection reagents, according to the manufacturer's guidelines. The hybridization signal was detected using Super Signal West Femto Maximum Sensitivity Substrate (Thermo Scientific).

### Construction of complemented strains

DNA fragments containing the *sigD* gene alone or with genes downstream of *sigD* (from *CD0267* to *CD0272*) were generated by PCR from genomic DNA of *C. difficile* strain 630∆erm using *sigD*comptetF-*sigD*comptetR and *sigD*comptetF-*CD0272*comptetR primers, respectively (Table S1). The PCR products were then cloned into pRPF185 digested by SacI and BamHI placing genes under control of a tetracycline inducible promoter [25]. Using the E. coli HB101 (RP4) as donor, plasmids were transferred by conjugation into the C. difficile 630Δerm sigD mutant, giving the sigD::erm + pRPF-sigD and sigD::erm + pRPF-sigD to CD0272 strains.

### Microarray design for the *C. difficile* 630genome, DNA-array hybridization and data analysis

The *C. difficile* 630 genome was obtained from EMBL database. Probe design for the microarray was performed by using the OligoArray 2.0 software[26]. One or 2 oligonucleotides were designed for each 3785 genes (we were unable to design oligonucleotides for 28 genes) and the microarrays were produced by Agilent. Probes were replicated twice on the array to reach a final density of 14224 probes per array. Five hundred thirty-six positive controls and 984 negative controls were also included. The description of the microarray design was submitted to the GEO database (accession number GPL10556). Total RNA was extracted from cells of 4 independent cultures for each growth condition. The cDNAs were labeled with either Cy3 or Cy5 fluorescent dye (GE Healthcare, Little Chalfont, UK) using the SuperScript Indirect cDNA labeling kit (Invitrogen) as previously described [27].

A mixture of 5µg of RNA and 1µg hexanucleotide primers (pd(N)6 Roche) was heated to 70°C for 5 min and quicky chilled on ice. We then sequentially added: 1X first-strand buffer, dithiothreitol (20mM), dNTP mix, Rnase OUT and 1600 units of Superscript III reverse transcriptase in a total volume of 24µl. The reaction was incubated 3h at 42°C to generate cDNAs. After alkaline hydrolysis and neutralization, cDNAs were purified on SNAP columns (Invitrogen) and precipitated with ethanol. The cDNAs were then mixed with Cy3 or Cy5 dyes (GE healthcare), incubated 1 h at room temperature in the dark, and purified on SNAP columns. 200 pmol of Cy3 and Cy5-labeled cDNAs was mixed and concentrated with microcon (Millipore). Hybridization was performed in micro-chambers for 17 h at 65°C according to the manufacturer’s recommendations. 8 differential hybridizations were performed and each RNA preparation was hybridized with a dye switch. The array was then washed successively with Gene Expression Wash Buffer 1 and 2 (Agilent). We realized arrays scanning with a GenePix Pro 6 dual-channel (635 nm and 532 nm) laser scanner (GenePix). All data were analyzed with R and Limma (Linear Model for Microarray Data) software from the Bioconductor project (www.bioconductor.org). The background was corrected with the “Normexp” method [28], resulting in strictly positive values and reducing variability in the log ratios for genes with low levels of hybridization signal. Then, we normalized each slide with the ‘Loess’ method [29]. In order to identify genes differentially expressed, we used the bayesian adjusted *t*-statistics and performed a multiple testing correction of Benjamini and Hochberg [30] based on the false discovery rate. A gene was considered as differentially expressed when the p-value is < 0.05. The complete experimental data set was deposited in the GEO database with the accession number GSE29275.

### Mapping of the transcriptional start sites by RACE-PCR

The initiation sites of transcription were determined from total RNA of *C. difficile* using the 3 '/ 5' RACE kit (Roche Diagnostics) for rapid amplification of cDNA ends as recommended by the manufacturer. The primers used are presented in Table S1.

### Overexpression of *flgM* in *C. difficile* 630*Δerm*

The promoter region of *CD2767* and the *flgM* ORF were amplified using primers P*2767*F-P*2767*R and primers *flgM*F-*flgM*R respectively (Table S1). Both PCR products were then digested by EcoRI and ligated with each other. Ligation product was amplified using primers P*2767F* and *flgM*-R, digested and cloned into the XhoI and PvuI restriction sites of pMTL007. The resulting plasmid was transformed into *E. coli* HB101 (RP4) and then transferred via conjugation into *C. difficile* 630Δ*erm*, giving the 630Δ*erm* + pMTL::P*CD2767*-*flgM*.

### Overexpression of *tcdR* in *C. difficile* 630*Δerm* and in the *sigD* mutant

The *tcdR* gene with its own promoter region (-810 to +825 from the translational start site) was amplified by PCR using OS314 and OS315 primers (Table S1). The PCR fragment was cloned into the BamHI and HindIII sites of pMTL84121 [31] to produce plasmid pDIA5941. Using the E. coli HB101 (RP4) as donor, this plasmid was transferred by conjugation into both *C. difficile* 630∆*erm* and its derivative *sigD* mutant to give 630∆*erm* + pDIA5941 and the *sigD* mutant + pDIA5941.

### Cloning, expression, and purification of SigD-His-tagged and FlgM-His-tagged fusion proteins in *E. coli*

The pQE30 expression system (Qiagen) was used to overexpress the SigD and FlgM proteins in *E. coli* M15 pREP4 as N-terminal hexa-His-tagged proteins.DNA fragments (obtained with chromosomal DNA of *C. difficile* 630Δ*erm* as the template) containing the *sigD* or *flgM* gene was generated using *sigD*-surF-*sigD*-surR and *flgM*-surF-*flgM*-surR, respectively (Table S1). The PCR products were then cloned into XhoI and HindIII of pQE30. *E. coli* M15 competent cells were transformed with the resulting plasmids.

*E. coli* recombinant strains were grown at 37°C in LB medium containing ampicillin and kanamycin. Protein expression was achieved by induction with 1mM IPTG and a subsequent incubation of the culture for 4 h at 37°C. Cells were then harvested by centrifugation. The His-tagged proteins were purified by affinity chromatography on Ni^2+^-nitrilotriacetic acid agarose (Qiagen) using Poly-Prep columns (BioRad) according to the manufacturers’ recommendations. Polyclonal anti-SigD and anti-FlgM antibodies were obtained by BALB/c mouse immunization (agreement number BI/11-03-01/2; AgroBio).

### Western blot analyses

Total proteins were extracted from cultures in BHI or TY broth. *C. difficile* cells were harvested and washed in 20 mM Tris-HCl (pH 8.0) solution. The cells were then resuspended in 4% (w/v) SDS solution, shaken for 60 min and sonicated twice on ice for 1 min. Extractswere heated at 100°C for 5 min and centrifuged at 11,000 g for 5 min.

Proteins separated by SDS-PAGE were electroblotted onto Hybond-enhanced chemiluminescence (ECL) nitrocellulose membranes (4°C for 1 h, 100 V) (Amersham Biosciences). Membranes were probed first with mouse antisera to SigD (this study) , FlgM (this study) or TcdA (Santa Cruz biotechnology, inc), or with rabbit antisera to FliC or *B. subtilis* SigA provided by M. Fujita [32] used at dilution of 1:1000 (SigD, FlgM) or at 1:10000 (TcdA, FliC, SigA). Primary antibodies were detected using a HRP-conjugated sheep α-mouse (GE healthcare) or goat α-rabbit secondary antibody (Jackson Immuno Research) at a dilution of 1:10000. Immunodetection of proteins was performed with the SuperSignal West Femto kit (Thermo Scientific) according to the manufacturer's recommendations. Blots were exposed to CL-XPOSURE films (Thermo Scientific) and developed.

### Gel retardation experiments

Fragment of 249 bp containing the *tcdR* promoter was amplified by PCR from genomic DNA of *C. difficile* 630 strain with primers *tcdRup*-F and *tcdRup*-R. For the radioactive labelling of the *PtcdR* PCR fragment, *tcdRup*-F primer was end-labelled with T4 polynucleotide kinase (Fermentas) and γ32-P-adenosine triphosphate (3000Ci.mM^-1^; Perkin Elmer) as recommended by the manufacturer. After PCR, amplified labelled fragment was then purified by QIAquick Nucleotide Removal kit (Qiagen^TM^). *E. coli* RNA polymerase holoenzyme and core enzyme forms were purchased from Epicenter. The labeled fragment (0.2 nM) was incubated for 60 min at room temperature in 10 µl of glutamate buffer [6] containing SigD purified, *E. coli* σ^70^ RNA polymerase holoenzyme, *E. coli* RNA polymerase core enzyme or *E. coli* RNA polymerase core enzyme preincubated with a four-fold molar excess of SigD. Four microliters of a heparin-dye solution (150 mg of heparin per ml, 0.1% bromophenol blue, 50% sucrose) in glutamate buffer was added and the mixture was loaded during electrophoresis on a 4.5% polyacrylamide gel prepared in Tris-borate-EDTA buffer [6]. After electrophoresis (2 h at 13 V/cm), the gel was dried, transferred to filter paper, and analyzed by autoradiography.

### Relative quantification of toxin expression

Total toxin amounts were quantified in supernatants from TY cultures using the commercial RIDASCREEN^®^-ELISA (R-Biopharm) as previously described and according to the manufacture’s protocol [8,11].

### Motility assays

Motility assays were performed using BHI motility agar tubes (0.175% agar), inoculated and grown anaerobically for 24 hours at 37 °C, as previously described [33].

### Triton X-100 autolysis assay

*C. difficile* cultures grown until exponential, late exponential or stationary phases were harvested, washed twice, and resuspended in 50 mM potassium phosphate buffer (pH 7.0) containing 0.01% of Triton X-100 (Triton X-100 acts as a nonionic detergent that forms micelles with lipoteichoic acids known to inhibit the autolytic activity in the peptidoglycan). The cells were then incubated anaerobically at 37 °C and the lysis monitored by measuring the absorbance at O.D. 600 nm at regular time intervals (Ultraspec 1100 Pro, Amersham Biosciences).

## Results

### Impact of *sigD* inactivation in *C. difficile* 630∆*erm*

The *C. difficile* 630 genome encodes putative SigD (*CD0266*) and anti-SigD (*CD0229*) factors homologous to SigD and FlgM of *B. subtilis*, with 34% and 43% identity, respectively. Both *sigD* and *flgM* genes are located in the region encoding flagellar apparatus [19]. To analyze the global role of SigD in *C. difficile*, we inactivated the *sigD* gene in *C. difficile* 630*∆erm* using the Clostron system [24]. Insertion of the group II intron into the target gene was verified by PCR using *sigD* and intron specific primers (Table S1, Figure S2). Moreover, Southern blot analysis confirmed that only one insertion occurred in the *sigD* mutant (Figure S2).

**Figure 1:**
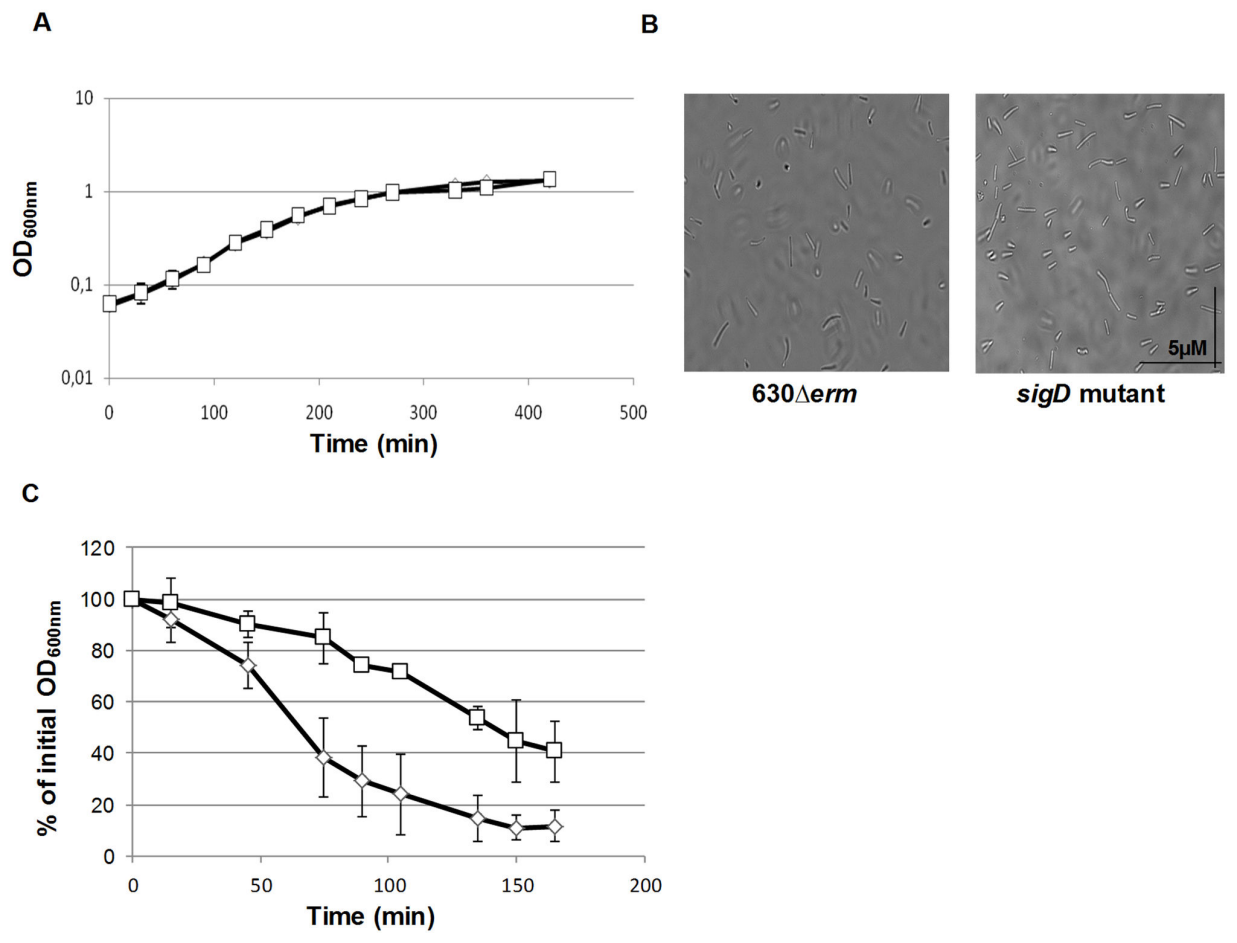
Phenotypic analysis of the *sigD* mutant. A: Growth curves in BHI medium showing no differences between *sigD* mutant (□) and 630*∆erm*strain (◊). B: Contrast phase microscopy during exponential phasein BHI medium showing the lack ofimpact of *sigD* inactivation on cells separation. C: Triton X-100 induced autolysis of 630*∆erm* (◊) and *sigD* mutant (□) strains at stationary phase showing that *sigD* mutant lyses more slowly than 630*∆erm*. The autolysis is expressed in percent initial absorbance at an optical density of 600 nm. Error bars indicate standard deviation.

We first analyzed the impact of *sigD* inactivation on growth and on autolysis of *C. difficile*, since SigD regulates autolysis in *B. subtilis* [23,34]. The inactivation of *sigD* had no effect on the growth kinetics of *C. difficile* in BHI medium (Figure 1A). In addition, as shown in phase contrast microscopy, the *sigD* mutant was not impaired in cell separation (Figure 1B). These results suggest, that unlike *B. subtilis*, SigD does not control expression of autolysins involved in cell separation during vegetative growth of *C. difficile*. We also explored the possible implication of SigD in global autolysis of *C. difficile* by performing Triton X-100 autolysis assays [35]. The wild-type and mutant strains did not show significant difference in autolysis at mid-and late exponential growth phases. However, the *sigD* mutant lysed at a slower rate compared to the wild type in stationary phase (Figure 1C). Meanwhile, as shown in a recent study [19], the *sigD* mutant also displayed a loss of motility and flagellin synthesis (see below). Thus, the inactivation of *sigD* in *C. difficile* impairs motility and decreases autolysis at the stationary phase, but does not impair cell septation during the vegetative growth phase. We also examined the sporulation and germination yields by following the development of heat-resistant colonies, but we observed no difference between the *sigD* mutant and wild-type strains. This result suggests that, like in *B. subtilis*, the contribution of SigD to sporulation, if any, is modest [36].

### Transcriptional and translational expression levels of *sigD*, *flgM* and *fliC* during growth phases of *C. difficile* 630∆*erm*

**Figure 2:**
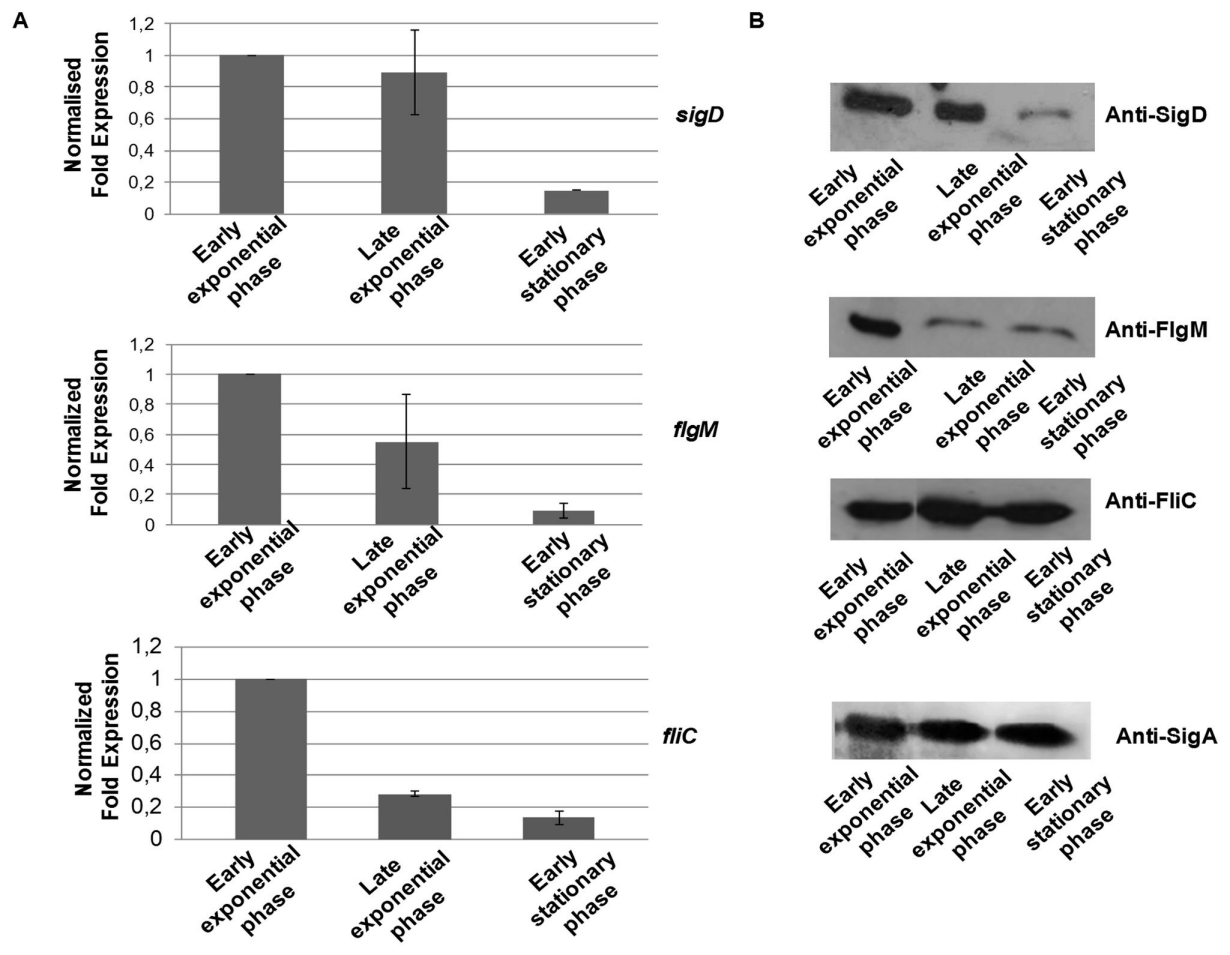
*sigD*, *flgM* and *fliC* transcriptional expression and protein level during growth in BHI medium. A: Quantitative RT-PCR analysis of *sigD*, *flgM* and *fliC* expression. Results are expressed as relative expression of *sigD*, *flgM* and *fliC* normalized by the 16S rRNA housekeeping gene. Error bars correspond to standard deviation from three biological replicates. B: Western blot analysis of SigD, FlgM and FliC protein levels. SigA antibodies were used as an internal control. The results are representative from at least three biological replicates.

In order to find appropriate growth conditions to study and to identify the SigD regulon, transcription of *sigD, flgM* (which encodes a putative anti-SigD factor) and *fliC* (which encodes flagellin) was analyzed by qRT-PCR during growth of *C. difficile 630∆erm* in BHI medium. The levels of transcription of *sigD* were similar at mid- and late exponential phases, but decreased at early stationary phase (Figure 2A). Consistent with the *sigD* transcriptional level we showed by Western blot experiments using anti-SigD antibodies, that the level of SigD protein is stable during the exponential phase and decreases at early stationary phase (Figure 2B). 

Transcription of *flgM* was maximal at early exponential phase and decreased from late exponential phase to reach the lowest level in stationary phase, which is also consistent with the level of the FlgM protein during the growth phases (Figure 2). Indeed, we showed by Western blot analysis using anti-FlgM antibodies that the level of FlgM was higher during exponential phase and decreased during late exponential and stationary phases of growth. Finally, although the transcriptional expression of *fliC* decreased along the growth, the level of FliC protein remained the same (Figure 2).

### Comparative transcriptomic analysis of gene expression profiles of *C. difficile* 630∆erm and the sigD mutant

Based on the expression kinetics of *sigD* and *flgM*, we decided to compare the expression profiles of the 630*∆erm* and the *sigD* mutant at the late exponential phase (i.e. 6 h of growth) in BHI medium. In total, 35 genes were up-regulated and 68 genes down-regulated in the *sigD* mutant when compared to the wild-type strain (*p*≤0.05). We observed that SigD regulates genes involved in various functions such as motility, membrane transport, metabolism, regulation and toxin synthesis (Table S2). To validate the transcriptomic profile data, we selected a subset of 20 genes related to various functions, and tested their transcription level by qRT-PCR (Table 2). qRT-PCR results and microarrays data exhibited high correlation coefficient (R2=0.88) (Table 2).

**Table 2:** Comparison of the expression of specific genes in microarrays and quantitative RT-PCR analysis between *C. difficile* 630*∆erm* and the *sigD* mutant.

**Genes**	**Name**	**Product**	**micro array**	**qRT-PCR**
*CD1304*	*acd*	Mannosyl-glycoprotein endo-beta-N-acetylglucosamidase	0.80	0.76
*CD1036*	*cwp17*	Putative N-acetylmuramoyl-L-alanine amidase, autolysin	0.69	1.10
*CD2767*	*cwp19*	Putative cell surface protein	0.78	0.59
*CD0211*	*licC*	CTP:phosphocholine cytidylyltransferase	0.05	0.026
*CD3527*		ABC-type transport system, iron-family ATP-binding protein	0.04	0.027
*CD0767*	*srlB*	PTS system, sorbitol-specific IIA component (Glucitol)	2.99	4.94
*CD0057*	*sigH*	RNA polymerase factor sigma-70	1.00	1.18
*CD2214*	*sinR*	Transcriptional regulator, HTH-type	5.65	4.27
*CD2215*		Transcriptional regulator, HTH-type	3.75	3.29
*CD0618*		Transcriptional regulator, LytR family	3.88	2.63
*CD1214*	*Spo0A*	Stage 0 sporulation protein A	1.00	1.03
*CD0266*	*sigD*	RNA polymerase sigma-28factor for flagellar operon	0.13	0.04
*CD0229*	*flgM*	Negative regulator of flagellin synthesis (Anti-sigma-d factor)	0.02	0.007
*CD0239*	*fliC*	Flagellin C	0.02	0.0007
*CD0240*		Glycosyltransferase	0.06	0.008
*CD0244*		Putative CDP-glycerol:Poly(glycerophosphate) glycerophosphotransferase	0.04	0.044
*CD0663*	*tcdA*	Toxin A	0.24	0.122
*CD0660*	*tcdB*	Toxin B	1.00	0.17
*CD0659*	*tcdR*	Alternative RNA polymerase sigma factor	0.54	0.07
*CD0661*	*tcdE*	Holin-like pore-forming protein	1.00	0.44
*CD0664*	*tcdC*	Negative regulator of toxin gene expression	1.00	0.64

The microarray data highlighted that most of the motility genes were controlled by SigD, as observed in *B. subtilis* [37]. Indeed, the expression of most genes encoding flagellar hook-associated proteins as well as the flagellin and the flagellum cap protein (*CD0226* to *CD0240*) and the expression of the flagellar glycosylation genes (*CD0241* to *CD0244*) (Figure S1) was highly decreased in the *sigD* mutant (magnitude of change ranged from 11-fold to 50-fold) (Table S2). We confirmed by Western blot analysis that FliC was not detected in the *sigD* mutant (see below), as described previously [19] and that is consistent with the absence of *fliC* gene transcription (Table S2) and the loss of motility in the *sigD* mutant (see below). The expression of most genes encoding the hook basal body (*flgB to flgH*) (Figure S1) was only slightly decreased (magnitude of change ranged from 1.58-fold to 1.96-fold), suggesting that they could still be transcribed from another sigma factor. Actually, when RACE-PCR experiment was conducted to map a putative promoter upstream *flgB*, we identified a transcriptional start site located 261 nucleotides upstream of the starting codon of *flgB* with a consensus sequence probably recognized by SigA ( **A**T**A**ACA-N_17_- **C**ATAA**A** ) (divergent bases are in bold). Whereas expression of genes upstream *sigD* is slightly affected by the *sigD* mutation, genes directly downstram of *sigD* (*CD0267* to *CD0272*) (Figure S1) were found highly downregulated. However, no putative promoter sequence was found upstream of *CD0267* suggesting a probable polar effect of the *sigD* mutation on the expression of genes downstream of *sigD* (*CD0267* to *CD0272*). Finally, we observed that the expression of *flgM* (the putative anti-SigD factor) decreased (50-fold) in the *sigD* mutant (Table S2). Therefore we further investigated below the mechanism of the positive control of SigD on the expression of *flgM*.

Concerning cell wall proteins, the expression of *cbpA* encoding a surface exposed adhesion [38], *CD0514*, encoding a cell surface protein, and *CD0211*, encoding a CTP:phosphocholine citidylyltransferase decreased in the *sigD* mutant. Although SigD does not significantly regulate *CD1036* and *CD1304*, which encode cell wall autolysins, the expression of *CD0226*, encoding a putative lytic transglycosylase, decreased dramatically in the *sigD* mutant. Interestingly, lytic transglycosylases (enzymes degrading glycan chains of peptidoglycan) are considered to be autolytic [39] and have been recently shown as required for full motility of several Gram positive or Gram negative species [40].

Many genes encoding membrane transport associated proteins are differentially expressed in the *sigD* mutant (Table S2). For example, the expression of *CD3525-CD3527*, encoding putative ABC transport system proteins and *CD3373* and *CD3375*, encoding putative magnesium transporters, decreased in the *sigD* mutant. Conversely, the expression of *CD0206-CD0208* and *CD0764-CD0767*, encoding phosphotransferase sugar (PTS) transport systems of fructose and sorbitol-specific respectively, increased (Table S2). Several genes involved in the metabolism of amino acids were up-regulated in the *sigD* mutant, whereas genes involved in the metabolism of carbon, and nucleic acids were down-regulated (Table S2).

We observed that expression of several transcriptional regulators increased in the *sigD* mutant (Table S2). Among them we found *CD2214* encoding a SinR-like pleiotropic regulator, which controls biofilm formation and sporulation in *B. subtilis* [41,42], *CD0618*, which encodes a LytR-like autolysin regulator known in *Staphylococcus aureus* to affects autolysis [43] and *CD0616* encoding a transcriptional regulator of the MerR family, which includes regulators responding to oxidative stress, heavy metals or antibiotics [44]. It is interesting to note that expression of *spo0A*, encoding the global response regulator of the sporulation initiation [16], and of *sigE* (*CD2643*), *sigF* (*CD0772*), *sigG* (*CD2642*) and sigK (*CD1230*) genes, encoding sporulation sigma factors [45], was not modified unlike recently observed in a *sigH* mutant [18], and is consistent with the absence of effect of SigD on sporulation. Finally, expression of *CD1275* and *CD1064* encoding the global transcriptional regulators CodY and CcpA, respectively did not differ between wild type and *sigD* mutant strains. 

### Complementation of *sigD* mutation

**Figure 3:**
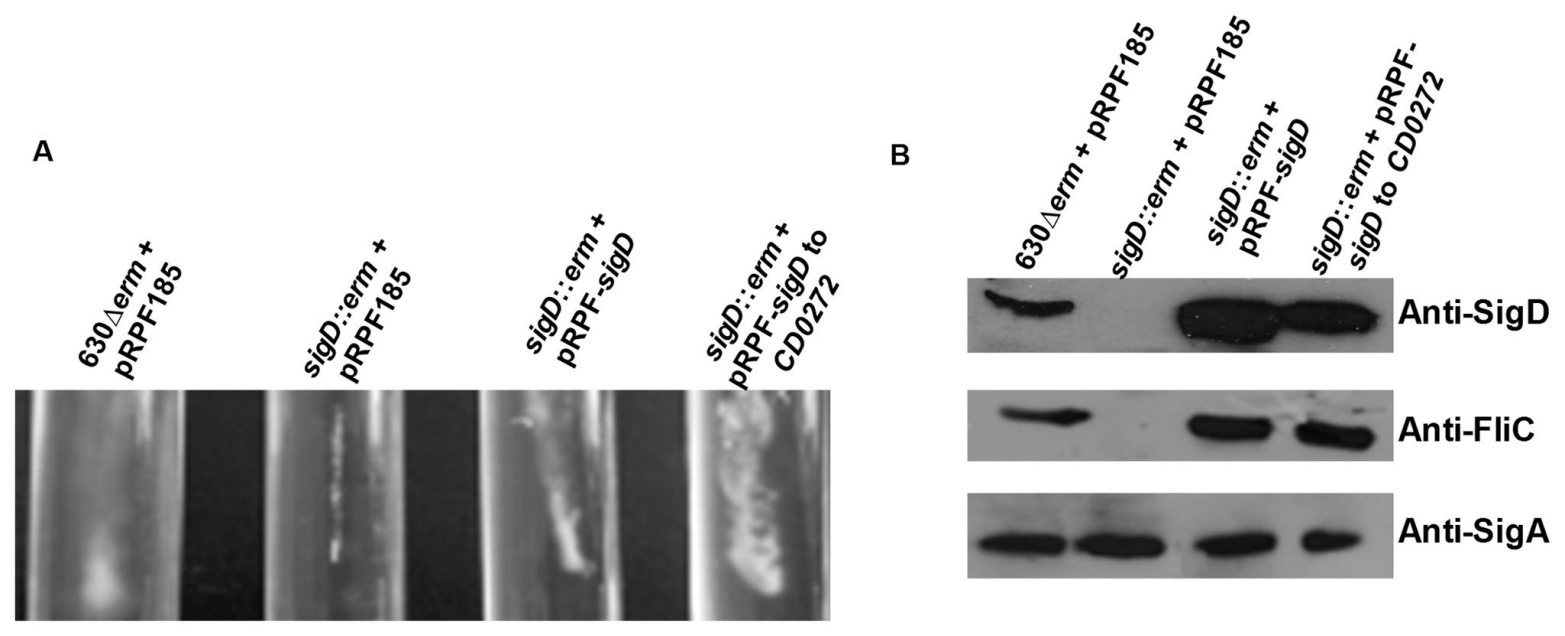
Effect of *sigD* complementation on motility and expression of SigD and FliC. A: Motility assays in agar soft tubes (0.175%) of *C. difficile* 630*∆erm* + pRPF185, *sigD*::*erm*+ pRPF185 and *sigD*::*erm* complemented with the pRPF-*sigD* or the pRPF-*sigD* to *CD0272*. B: SigD and FliC protein levels were estimated by Western Blot analysis on 630*∆erm* + pRPF185, *sigD*::*erm*+ pRPF185, and *sigD*::*erm* complemented with the pRPF-*sigD* or the pRPF-*sigD* to *CD0272*.SigA antibodies were used as an internal control. The results are representative from at least three biological replicates.

The *sigD* gene is located in the 3’ region of the large operon that encodes proteins constituting the hook basal body and starting with the *flgB* gene. To determine whether SigD is expressed independently from genes upstream, we performed a RACE-PCR experiment to localize a putative promoter of *sigD*. However we did not find transcriptional start upstream of *sigD*, suggesting that *sigD* is part of a larger operonic structure. Owing to the complex regulation of flagella expression and to confirm that the defect of motility was directly due to the disruption of *sigD*, the complementation of the *sigD* mutant was undertaken. For this purpose we constructed two plasmids, one carrying only the wild type *sigD* gene and another one carrying the wild type *sigD* plus genes downstream untill *CD0272* (Figure S1). We used a tetracycline inducible promoter ATc in both plasmids to control gene expression (see Experimental procedures). Both complemented strains were restored for SigD and FliC synthesis (Figure 3). Interestingly, the *sigD* complemented strain is partially restored for motility, whereas the *sigD*-*CD0272* complemented strain appears as motile as the wild-type strain, suggesting that the expression of genes downstream *sigD* seems to be required for full motility of *C. difficile* (Figure 3). Overall, these data strongly support evidence that SigD controls expression of flagellar genes in *C. difficile*.

### SigD modulates Paloc genes expression

**Figure 4:**
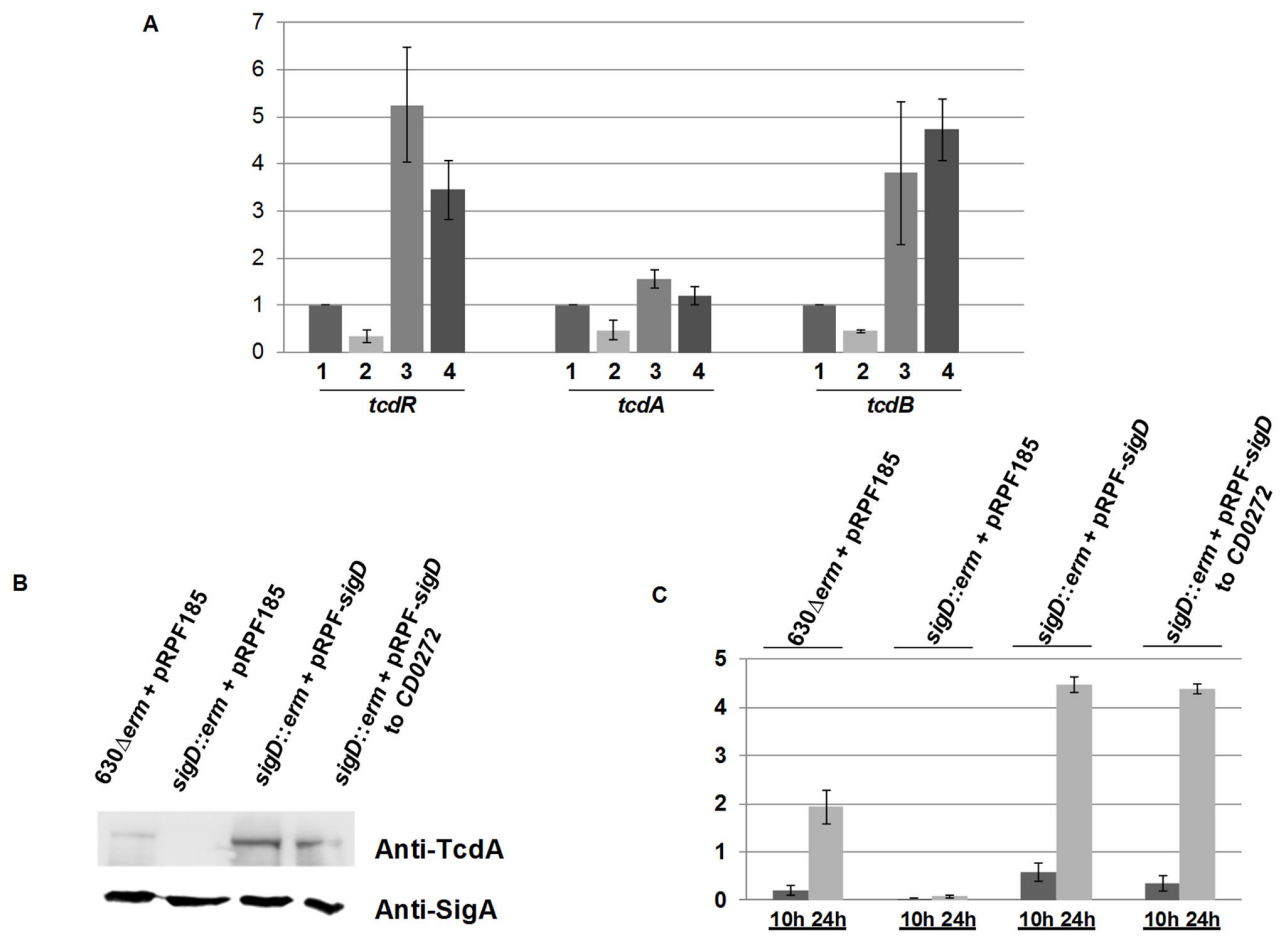
Effect of *sigD* inactivation on toxins expression during stationary phase. A: Quantitative RT-PCR analysis of *tcdA*, *tcdB* and *tcdR* expression in strains 1: 630*∆erm* + pRPF185, 2: *sigD*::*erm*+ pRPF185, 3: *sigD*::*erm* + pRPF-*sigD* and 4: *sigD*::*erm* + pRPF-*sigD* to *CD0272* grown in TY medium. Results are expressed as relative expression normalized by the 16S rRNA housekeeping gene. Error bars correspond to standard deviation from 3 biological replicates. B: Western blot analysis of TcdA from crude proteins extracts of *C. difficile* 630*∆erm* + pRPF185, *sigD*::*erm*+ pRPF185, *sigD*::*erm* + pRPF-*sigD* and *sigD*::*erm* + pRPF-*sigD* to *CD0272* strains grown in TY medium. SigA antibodies were used as an internal control. C: TcdA and TcdB expression levels in supernatants of *C. difficile* 630*∆erm* + pRPF185, *sigD*::*erm*+ pRPF185, *sigD*::*erm* + pRPF-*sigD* and *sigD*::*erm* + pRPF-*sigD* to *CD0272* Strains were quantified using ELISA test after 10 and 24 hours growth in TY medium. Error bars correspond to standard deviation from at least three biological replicates.

The transcriptomic analysis showed a decrease of *tcdA* and *tcdR* expression (4.16-fold and 1.85-fold, respectively) in the *sigD* mutant compared to the wild type grown in glucose-containing BHI medium (Table S2). We did not see differences in *tcdB* expression between the wild-type and the *sigD* mutant strains, probably due to the low level of *tcdB* transcripts at 6 hours of growth, as previously observed [46]. However, when we further analyzed by qRT-PCR the expression of the PaLoc genes in the wild type and the *sigD* mutant, we found that, in addition to *tcdA* and *tcdR* expression, the expression of *tcdB* also decreased in the *sigD* mutant grown in BHI medium (8.13-, 13.76- and 5.87- fold respectively) (Table 2). Furthermore, the same effect of *sigD* mutation on the Paloc genes transcription was observed in the optimal growth conditions for *C. difficile* toxin production, i.e. when cells are grown in glucose-free TY medium at the stationary phase (Figure 4A). Western blot analysis of crude extracts, using antibodies raised against TcdA (Figure 4B) and ELISA quantification of toxins A and B in the supernatant of 10 and 24 hours cultures (Figure 4C) confirmed the loss of toxin synthesis in the *sigD* mutant. As complementation of the *sigD* mutant by both SigD-expressing plasmids restore toxin genes expression and production (Figure 4). Taken together, these data indicate that SigD positively controls the expression of C. difficile toxin genes, as recently suggested by several groups [19,47], whereas the mode of action of SigD was not described. Therefore we further investigated the mechanism of this regulation (see below).

### Identiﬁcation of direct target genes of SigD

In the transcriptome analysis, 68 genes showed decreased expression in the *sigD* mutant, indicating that SigD exerts direct or indirect positive control on these genes in the wild-type. To find potential direct target genes controlled by SigD, we looked for the presence of the consensus sequence of *B. subtilis* SigD-dependent promoters (TAAA-N_13-16_GCC#G#ATAW) in the 300 bp region upstream of start codons of *C. difficile* genes using the GenoList web server (http://genodb.pasteur.fr/cgibin/WebObjects/GenoList), allowing three mismatches. Among the genes found to contain a *B. subtilis* SigD-like consensus sequence in their promoter regions, only 11 genes and operons are significantly and positively regulated by SigD, as observed in the comparative transcriptomic analysis (Table S2). This includes 5 late flagellar genes and 2 early flagellar genes, suggesting that multiple *sigD*-dependent promoters are implicated in the expression of the flagella regulon (Table S2, Table 3).

**Table 3:** *C. difficile* genes which expression significantly decreased by SigD and displaying a SigD consensus sequence in their promoter region.

**Gene**	**Function**	**Expression ratio** ***sigD* mutant/630*∆erm***	**Consensus sequence**
*CD0226*	putative lytic transglycosylase	0.08	aTAAAtattttttttatttatCCGATAAt
*flgM*	negative regulator of flagellin synthesis (anti-SigD factor)	0.02	aTAAAtatttttcttctttgaGCGATAAt
*flgK*	flagellar hook-associated protein FlgK (or HAP1)	0.03	aTAAAgaaagaacttattttcACGAAAAa
*fliC*	flagellin C	0.02	aTAAAgttatagattaacttgtCCGATAAt
*motA*	flagellar motor rotation protein MotA	0.53	aTAAAtgtaggttatattaggaGCGAAAAa
*CD0230*	putative flagellar biosynthesis protein	0.05	cTAAAaaaatgatagaggagatGCGAGGAt
*fliQ*	FliQflagellar biosynthetic protein	0.51	tTAAAagaaaagaaattaacTCGTGAAa
*tcdR*	toxin transcriptional regulator	0.53	aTAAAatttaatttatttgCCGATTAt
*CD2668*	transcription antiterminator, LicT family	0.45	aTAAAttgaatacaatatataaGCGTTAAc
*CD3028*	putative phosphosugar isomerase	0.43	tTAAAgagaatcttaaatatACGATTGa
*CD3527*	putative iron ABC transporter, ATP-binding protein	0.04	aTAAAgtaaataaattattgaGCGATTAt

**Figure 5:**
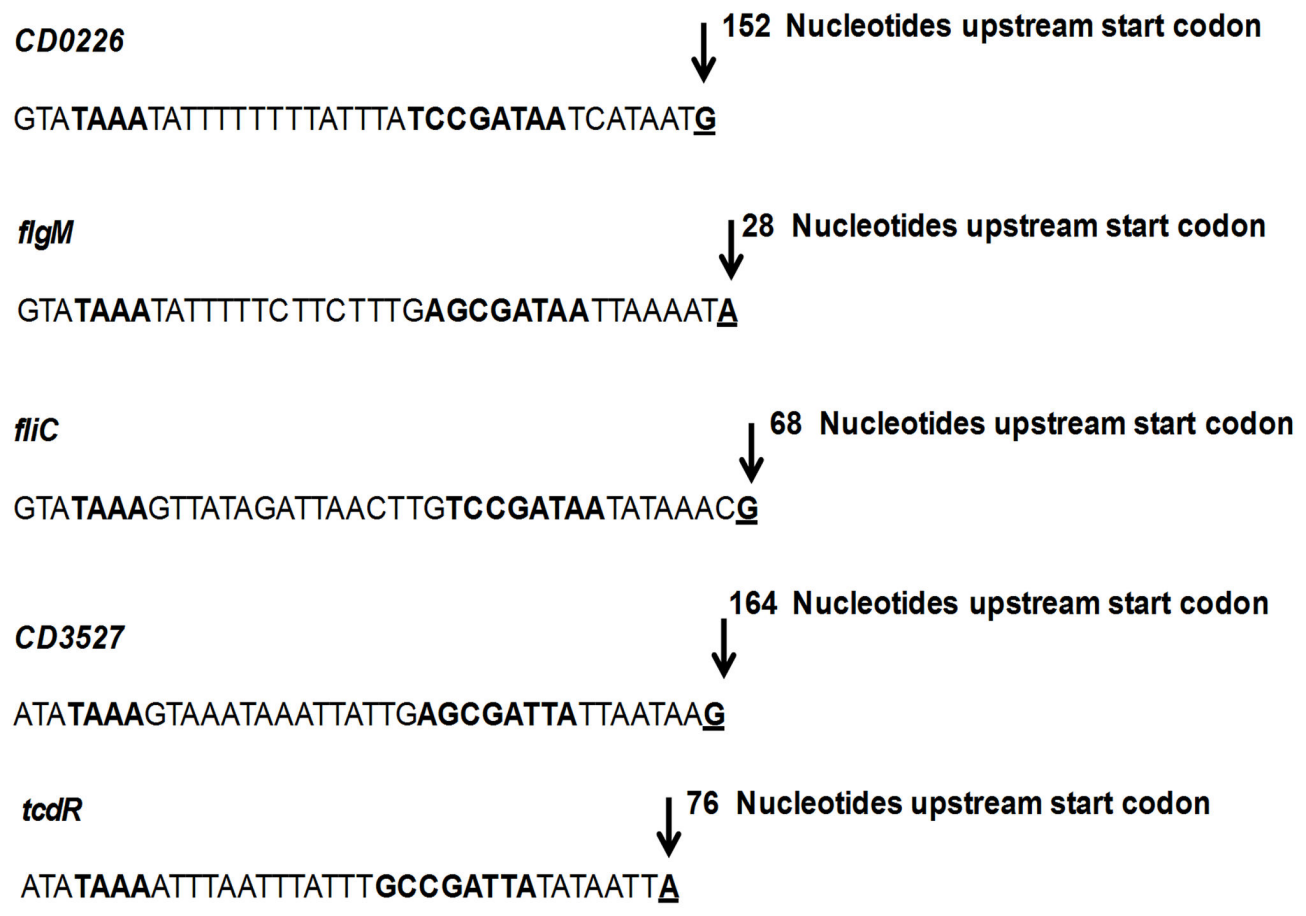
Identification of the SigD-dependent promoter sequence by RACE-PCR. SigD-dependent transcription start sites upstream of start codons of genes involved in motility (*CD0226*, *flgM, fliC*), membrane transport (CD3527) and virulence (tcdR). The transcriptional start sites are indicated in bold and underlined. The -35 and -10 boxes corresponding to SigD-dependent promoters are indicated in bold.

RACE-PCR experiments were then performed to confirm the promoter sequences for 5 out of the 11 genes identified. We found a transcription initiation site located 28 nucleotides upstream of the *flgM* start codon (Figure 5, Figure S1), which displays a *B. subtilis* SigD-like consensus sequence in its promoter region. Direct control of *flgM* by SigD is consistent with the dramatic decrease of the *flgM* transcription in the *sigD* mutant (Table S2). We also identified transcription initiation sites located 152, 68 and 164 nucleotides upstream of the *CD0226*, *fliC* and *CD3527* start codons, respectively, with a *B. subtilis* SigD-like consensus sequence in their promoter regions (Figure 5 Figure S1). These results strongly suggest that *C. difficile* SigD directly controls the expression of these genes. Interestingly, we also found a *B. subtilis* SigD-like consensus sequence in the promoter region of the *tcdR* gene as recently proposed [47] (Table 3). Indeed, we identified by RACE-PCR a transcription initiation site located 76 nucleotides upstream of the *tcdR* start codon, which displays a consensus sequence of the *B. subtilis* SigD-dependent promoters (TAAA –N_13_-GCCGAT**T**A) (divergent base is in bold) (Figure 5).

**Figure 6:**
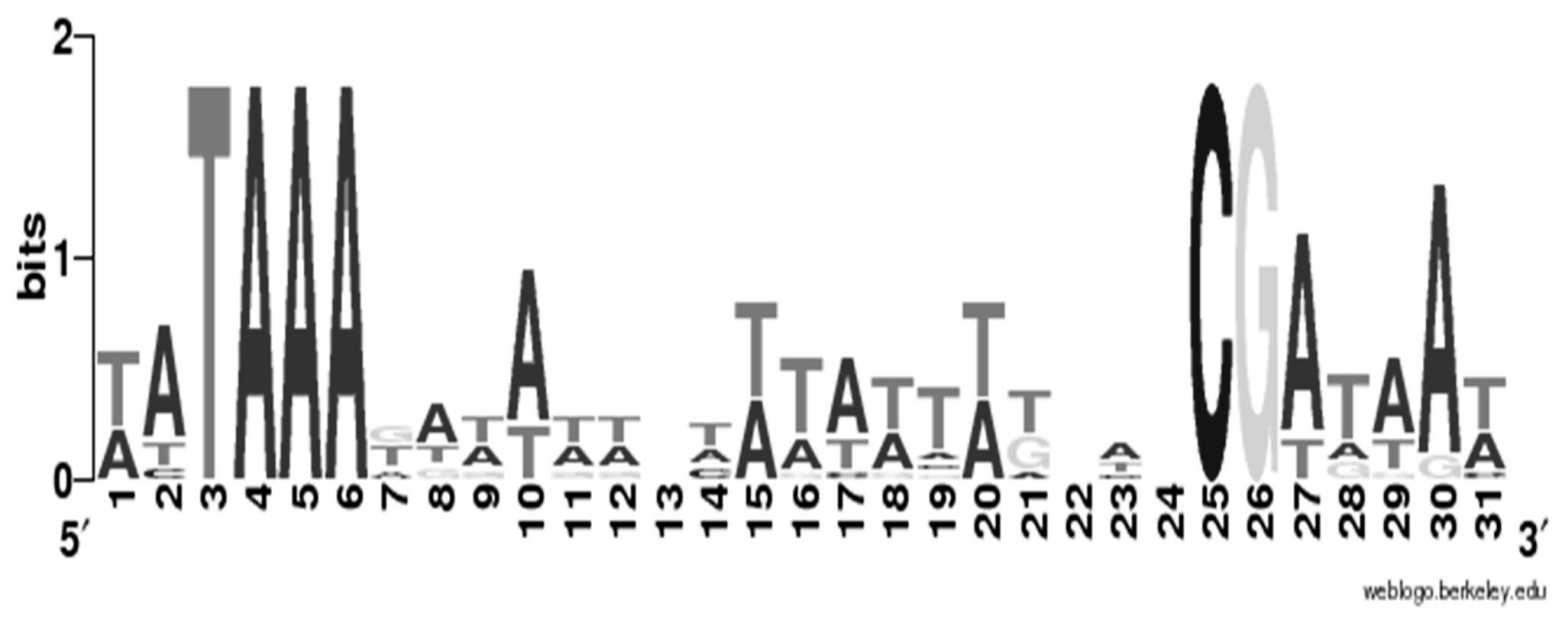
Consensus sequence of SigD-dependent promoters in *C. difficile*. The sequences of the direct genes listed in Table 3 were aligned using ClustalW. This sequence was obtained on the WebLogo website (http://weblogo.berkeley.edu). The height of the letters is proportional to their frequency.

The alignment of all probable SigD-dependent promoters using the WebLogo website (http://weblogo.berkeley.edu) and listed in Table 3, allowed to propose a consensus sequence of *C. difficile* SigD-dependent promoters, which contains two conserved motifs TAAA and CG separated by 15 to 18 bases (Figure 6). Surprinsingly, when we used the consensus sequence of *C. difficile* SigD-dependent promoters to found more genes under direct control of SigD in the *C. difficile* 630 genome, we did not find more than the eleven genes and operons previously cited in table 3.

Since, *C. difficile* SigD-dependent promoter sequence was only found in the promoter regions of *tcdR* and not in *tcdA* and *tcdB* promoter regions, the decreased expression of *tcdA*, *tcdB* and *tcdR* in the *sigD* mutant suggests that the regulation of toxin genes by SigD must be controlled via TcdR.

### SigD directly controls *tcdR* transcription

**Figure 7:**
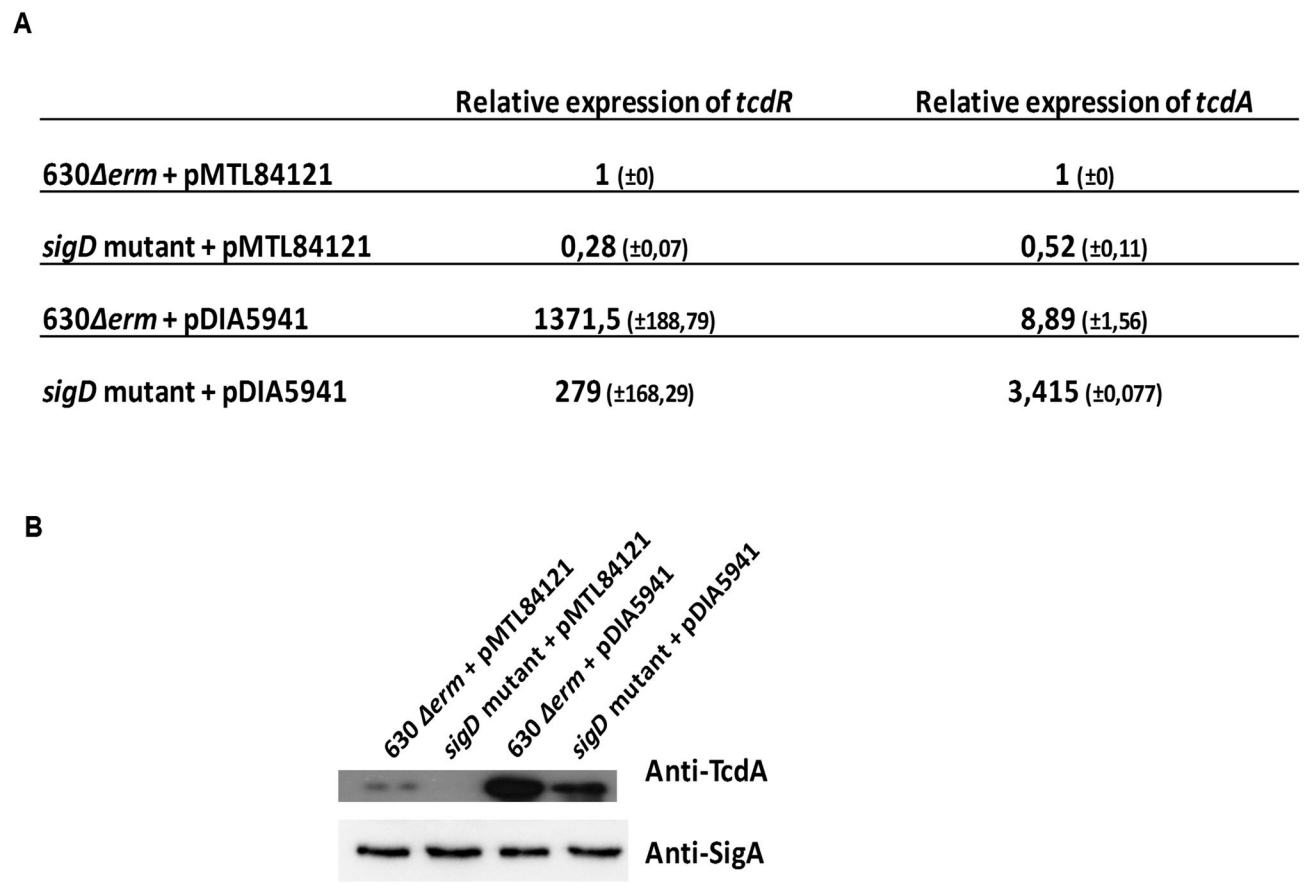
SigD controls *tcdR* transcription. A: Quantitative RT-PCR analysis of *tcdR* and *tcdA* expression in *C. difficile* 630*∆erm* + pRPF185, *sigD*::*erm*+ pRPF185 and *sigD*::*erm* complemented with the pRPF-*sigD* or the pRPF-*sigD* to *CD0272*, grown in TY medium. Results are expressed as relative expression normalized by the 16S rRNA housekeeping gene. B: TcdA protein level was estimated from crude proteins extracts of the *C. difficile* 630∆*erm*+pMTL84121, *sigD* mutant+pMTL84121, *C. difficile* 630∆*erm* + pDIA5941 and *sigD* mutant+ pDIA5941 grown in TY medium by Western blot analysis. SigA antibodies were used as an internal control. The results are representative from at least three biological replicates.

To determine whether SigD directly control *tcdR* transcription, a plasmid containing the *tcdR* gene with its promoter region (pDIA5941) was introduced into both 630*∆erm* and *sigD* mutant strains (see Experimental procedures). As expected, transcriptional analysis showed an overexpression of *tcdR* in both strains containing pDIA5941 (Figure 7A). However, the pDIA5941-containing 630∆*erm* strain expressing SigD, displayed a higher level (4.9 fold) of *tcdR* than the pDIA5941-containing *sigD* mutant (Figure 7A), confirming that SigD controls positively *tcdR* expression. We noted that difference of *tcdA* expression is lesser than that of *tcdR* expression in the pDIA5941-containing *sigD* mutant when compared to the pDIA5941-containing 630∆*erm* strain. This result is consistent with the fact that expression of *tcdA* is not directly linked to SigD but through the TcdR sigma factor (Figure 7A, 7B). Thus, SigD positively controls toxin gene expression by directly regulating *tcdR* transcription likely via the SigD-dependent promoter sequence present upstream of the promoter region of *tcdR*.

### RNA polymerase containing SigD binds specifically to *tcdR* promoter region

**Figure 8:**
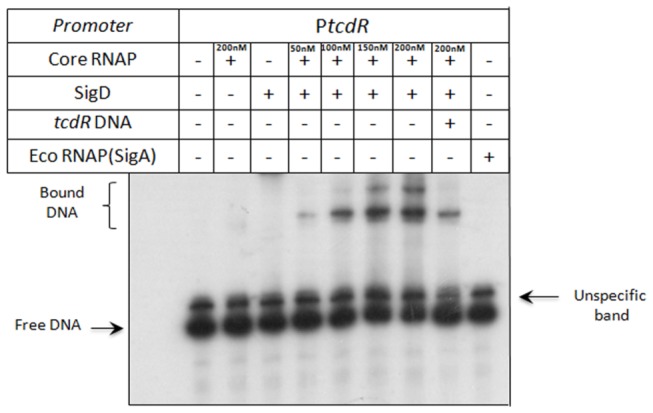
Gel mobility retardation of *tcdR* promoters with *E. coli* RNA polymerase core enzyme and SigD. A DNA fragment containing the *C. difficile* *tcdR* promoter region (P*tcdR*) was incubated with SigD, *E. coli* SigA RNA polymerase (200nM) or *E. coli* RNA polymerase core enzyme alone (200nM) or after pre-incubation with SigD protein. Increasing concentrations of RNA polymerase containing SigD are indicated in the figure (from 50 to 200nM).

Generally, Sigma factors like SigD are sequence-specific, DNA-binding subunits of RNA polymerase, ensuring the recognition of appropriate promote sites. Thus to determine whether RNA polymerase containing SigD activates *tcdR* transcription, we performed a gel mobility shift assay with the *tcdR* promoter DNA fragment and the RNA polymerase core enzyme purified from *E. coli* (Epicentre) with or without addition of SigD and challenged the complexes with heparin. Neither core enzyme nor SigD alone was able to shift the mobility of the *tcdR* promoter-containing fragment (Figure 8). However, when we mixed SigD with the core enzyme, the reconstituted RNA polymerase is able to form heparin-resistant complex at the *tcdR* promoter in a dose-dependent manner (Figure 8). The RNA polymerase containing the major vegetative sigma factor SigA was unable to the bind to the promoter region of *tcdR* (Figure 8). Moreover, the addition of an excess of unlabelled heterologous DNA [1 mg of poly (dI-dC)] did not prevent DNA binding (data not shown), while the addition of an excess of unlabeled homologous DNA effectively prevented DNA binding (Figure 8). Thus, it is clear that sigD directly activates *tcdR* expression by directing RNA polymerase core enzyme to recognize *tcdR* promoter and activate its transcription. 

### FlgM turns off the positive regulation of SigD on flagella and toxins expression

**Figure 9:**
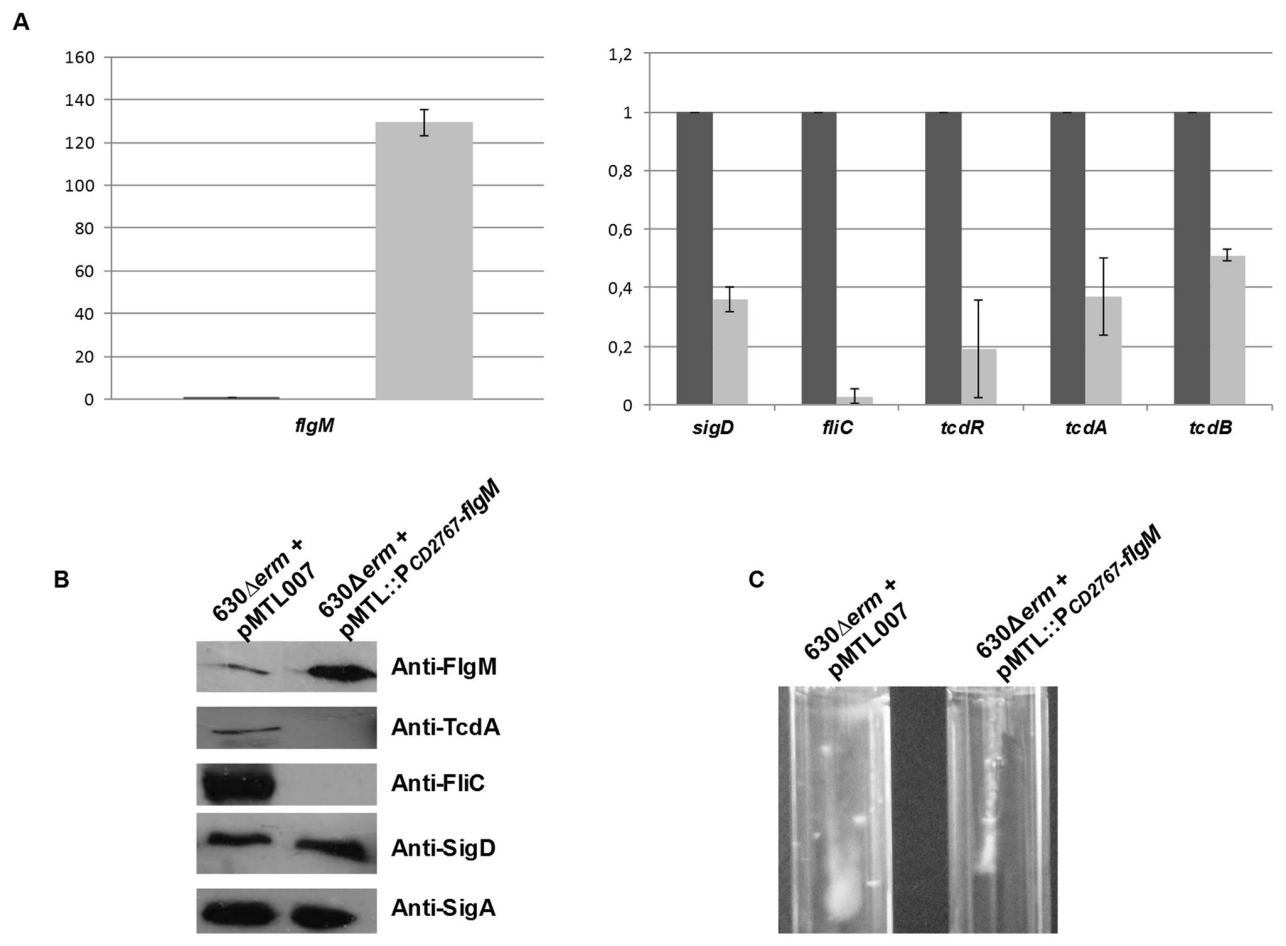
Effect of *flgM* overexpression on motility, flagellar and toxin genes expression. A: Quantitative RT-PCR analysis of *flgM, sigD*, *fliC*, *tcdR*, *tcdA* and *tcdB* expression was performed in *C. difficile* 630∆*erm*+pMTL007 and *C. difficile* 630Δ*erm* + pMTL::P*CD2767*-*flgM*, grown in BHI medium. Results are expressed as relative expression normalized by the 16S rRNA housekeeping gene. Error bars correspond to standard deviation from at least 3 biological replicates. B: Western blot analysis of FlgM, TcdA, FliC and SigD proteins from crude extracts of *C. difficile* 630∆*erm* + pMTL007 and *C. difficile* 630Δ*erm* + pMTL::P*CD2767*-*flgM*. SigA antibodies were used as an internal control. The results are representative from at least three biological replicates. C: Motility assay in agar soft tubes (0.175%) showing the loss of motility following *flgM* overexpression.

To support that SigD act as an alternative sigma factor on the positive regulation of flagella and toxins expression, we investigated the role of FlgM, the putative anti-SigD. In *B. subtilis* and *Salmonella typhimurium*, FlgM binds to SigD, thereby inhibiting premature expression of late flagellar gene [48,49]. We first tried to inactivate the *flgM* gene using the Clostron system, but repetitive attempts using different intron sites remained unsuccessful. Instead, *flgM* was overexpressed in the 630∆*erm* strain by cloning the *flgM* gene downstream of the *CD2767* promoter (under the control of the domestic sigma factor SigA; unpublished data) in pMTL007. Overexpression of *flgM* (130-folds) led to a decrease of the *sigD* expression (Figure 9B), indicated that FlgM interferes with the SigD protein to initiate transcription from its promoters, ie SigD-dependent *fliQ* promoter located 5 genes upstream. Moreover, although SigD is still present at a significant level, overexpression of FlgM leads to a complete loss of motility in the corresponding strain, which is related to the absence of *fliC* transcription and flagellin production (Figure 9). In addition, transcriptional analysis revealed that the expression of *tcdR*, *tcdA* and *tcdB* was also decreased in the presence of high level of FlgM (Figure 9A) and consequently on TcdA production as confirmed by a Western blot analysis (Figure 9B). Thus, overexpressed FlgM leads to a down-regulation of genes under positive control of SigD, and strongly support that SigD act as a sigma factor on the flagella and toxin genes expression.

## Discussion

Among Gram-positive bacteria, the regulatory properties of the SigD factor have been extensively studied in *B. Subtilis* where it controls flagellar synthesis, motility and vegetative autolysins [23,34,50]. The aim of our study was to characterize the regulatory properties of SigD in *C. difficile*, by comparing phenotypic properties and transcriptomic profiles of *C. difficile* 630∆*erm* and its *sigD* mutant.

In *B. subtilis*, SigD has been shown to play a critical role in the cell separation. Indeed, the major autolysins LytC, LytD and LytF [23,51] are under transcriptional control of SigD. Consequently, a *sigD* mutant does not form separate cells and grows constituvely in chains. In *C. difficile*, the inactivation of *sigD* does not have any impact on cell separation but a significant decreased autolysis is observed at the stationary phase. Among the 37 putative peptidoglycan hydrolases identified on the genome of *C. difficile* [52], only the genes *CD2141*, encoding a putative D-Ala-DAla carboxypeptidase, and *CD0226*, encoding a putative lytic transglycosylase have been shown transcriptionally deregulated in the microarray analysis. However, *CD2141* is upregulated in the *sigD* mutant strain and carboxypeptidases are known to not destroy the peptidoglycan mesh and are generally considered as peptidoglycan maturation enzymes [53]. Conversely, *CD0226* is downregulated in the *sigD* mutant. Transglycosylases are not true hydrolases because they cleave the glycosidic bond with a concomitant intramolecular transglycosylation reaction, but they are able to act as autolysins [39]. Furthermore, a SigD consensus sequence was identified in the promoter region of *CD0226*. Thus, control of *CD0226* by SigD could explain the lysis defect in the *sigD* mutant. Nevertheless, unlike *B. subtilis*, the role of SigD of *C. difficile* in the control of the autolysins appears to be very limited.

Recently, a link was established between transglycosylase activity and motility of *Helicobacter pylori* and *Salmonella typhimurium*, and between glucosaminidase activity and motility in *Listeria monocytogenes* [40]. Indeed, proper anchoring and functiounality of the flagellar motor could involve the maturation of the surrounding peptidoglycan by a hydrolytic enzyme. Interestingly, *CD0226*, encoding a putative lytic transglycosylase is the first gene of the late-stage flagellar genes. Further analysis should explore if a similar link exists in *C. difficile*.

Control of motility by SigD has been studied and demonstrated both in Gram negative (where it is usually called FliA), such as *Escherichia coli* [54] or *Salmonella typhimurium* [55] and in Gram positive bacteria, such as *Bacillus subtilis* [51]. Very recently, SigD has also been shown implicated in the positive regulation of motility in *C. difficile* [19] that is widely confirmed in this present study. Moreover, our microarray analysis combined to the identification of promoters regions by RACE-PCR and by in-silico analysis allows us to bring new elements on the transcription initiation of *sigD* and the flagellar regulon. First, transcriptional analysis shows that *sigD* inactivation in *C. difficile* affects only slightly the expression of genes encoding the hook basal body (early flagellar genes). This is consistent with the SigA-like consensus sequence identified by RACE-PCR upstream of the starting codon of *flgB*, the first gene of this operonic structure, indicating that the expression of the early flagellar genes is partly independent of the expression of SigD. From our in-silico analysis, the first probable SigD-dependent promoter in this operon is located upstream *motA* and another one is then found upstream *fliQ* (Figure S1). In *B. subtilis*, the *fla*-*che* transcription unit resembles the early flagellar genes element of *C. difficile* and a SigA-dependent promoter *P*_*fla/che*_ has also been found upstream the first gene of the operon [56]. *P*_*fla/che*_ has been shown essential for expression of the *sigD* gene but, unlike *C. difficile*, a weak SigD-dependent promoter P_D-3_, dispensable for motility has also been identified upstream of the primary *P*_*fla/che*_ promoter [22,57]. Two others SigD-dependent promoters have also been found within the *fla*-*che* transcription unit [58] of *B. subtilis*, the P_*ylxF3*_ promoter governing partly the expression of *sigD* [59] and the P_*sigD*_ promoter, residing immediately upstream of *sigD* itself but its activity is not clearly demonstrated [22,58]. In contrast to the early-stage flagellar genes, transcription of the late-stage flagellar genes is strongly affected by the *sigD* inactivation. In agreement with this observation, RACE-PCR experiments led to the identification of a SigD-dependent promoter upstream *CD0226*, the first gene of this cluster, whereas no SigA-dependent promoter could be found. Moreover, two others SigD-dependent promoters were identified within this region, one upstream of *flgM* and the other upstream *fliC*. In support of this, we showed a complete loss of *fliC* and *flgM* transcription in the *sigD* mutant and a restoration of their expression expression after complementation of *sigD* mutation. This is similar to *B. subtilis*, where the *hag* gene encoding flagellin and the *flgM* gene possesse a SigD-dependent promoter and is transcribed by the SigD containing RNA polymerase [57,60]. The flagellar glycosylation genes cluster is located 717 bp downstream from *CD0240* (the last gene of the late-flagellar genes region) [19] and its transcriptional expression is also strongly doxnregulated in a *sigD* mutant. Yet, no SigD-dependent promoter could be identified immediately upstream or within this cluster by our *in-silico* analysis, suggesting that these genes are cotranscribed with *fliC* and *CD0240* from the SigD-dependent promoter residing upstream *fliC*.

In *B. subtilis*, the expression of *sigD* is necessary for the transcription of genes involved in flagellar synthesis and chemotaxis [59,61] and the SigD-dependent transcription of late flagellar genes is repressed by FlgM, the anti-SigD factor, through a post-translational control [49]. FlgM directly binds to SigD and antagonizes its activity in the early stage of growth [62]. However, when the formation of the hook basal body is completed, SigD is released due to the secretion of FlgM from the cells through the assembled flagellar motor structure and genes under SigD-dependency are then transcribed [63]. In *C. difficile*, the overexpression of *flgM* inhibited SigD activity and consequently suppressed, like in the *sigD* mutant, motility and flagellin expression. Thus, we confirm that SigD is a positive regulator of motility in *C. difficile*, and further show the role of FlgM as an anti-SigD factor participating in the flagellar regulation. Other studies will be undertaken in future in our lab to analyze the probable secretion of FlgM in the culture supernatant.

The inactivation of *sigD* decreases dramatically the expression of *tcdA*, *tcdB* and *tcdR* [19] and it has been recently shown that *sigD* expression is negatively regulated by increasing intracellular level of the second messenger cyclic diguanilate (c-di-GMP), which impacts the expression of toxin genes [64]. Indeed, the regulation of *C. difficile* toxin production by the level of c-di-GMP, via the control of SigD, was recently established and a mechanism for the SigD-dependent regulation of toxin expression has been proposed [47]. However, the mode of action of SigD on the regulation of *tcdR* expression was not experimentally determined. In our study, we demonstrated the regulation of toxin genes by SigD through TcdR. Moreover, a SigD-dependent promoter predicted by the in-silico analysis is present upstream of the 5’ region of *tcdR* and has been confirmed by RACE-PCR. Most importantly, electrophoretic mobility shifts assays demonstrated the direct binding of SigD-containing RNA polymerase to the *tcdR* promoter. Therefore, this is the first study that unambiguously demonstrates the role of SigD in the controls of toxin synthesis via a direct regulation of the *tcdR* promoter. Thus SigD, which has never been reported as a positive regulator of toxin synthesis in other bacteria, appears as a key positive regulator of both motility and toxin synthesis in *C. difficile*.

## Supporting Information

Figure S1 **(adapted from Aubry et al [19])** : Flagellar locus from *C. difficile* 630, with location of the three SigD promoter sites identified by RACE-PCR (arrows above the flagellar locus). Dashed arrows indicate genes which posses a SigD consensus sequence and which significantly regulated by SigD. White triangle: mutagenesis of *sigD* gene using the Clostron system.
(TIF)

Figure S2 **Inactivation of *sigD* gene.** **A**: Schematic presentation of pMTL-based knocks-out plasmid. **a**: parental plasmid pMTL007. **b**: wild-type target gene. **c**: mutated target gene. Group II intron (black arrow), internal RAM conferring erythromycin resistance (white arrow) are represented.The locations of primers used for screening mutants are indicated. **B**: Confirmation of gene knockouts using PCR. Amplifications were performed on630Δ*erm* and630Δ*erm* *sigD::intron-erm* using: *sigD* target specific primers F and R (*sigD-F* and *sigD-R*), *sigD-F* and EBSu primers and ErmRAM-F and ErmRAM-Rprimers. **C**: Southern blot analysis of genomic DNA from *C. difficile* 630Δ*erm* and*C. difficile* 630Δ*erm* *sigD::intron-erm* with an intron probe. Chromosomal DNA (6µg in each reaction) was digested with HindIII.
(TIF)

Table S1 **Oligonucleotides used in this study.**
(DOCX)

Table S2 **Genes positively or negatively controlled by SigD according to the expression ratio in transcriptomic analysis of *sigD* mutant/strain 630*∆erm* after 6h of growth.**
(DOCX)
