# Supplementary figures and images for: Characterization of the SigD Regulon of C. difficile and Its Positive Control of Toxin Production through the Regulation of tcdR
Source: PLoS One. 2013 Dec 16;8(12):e83748. doi: 10.1371/journal.pone.0083748 (PMC3865298; doi:10.1371/journal.pone.0083748)

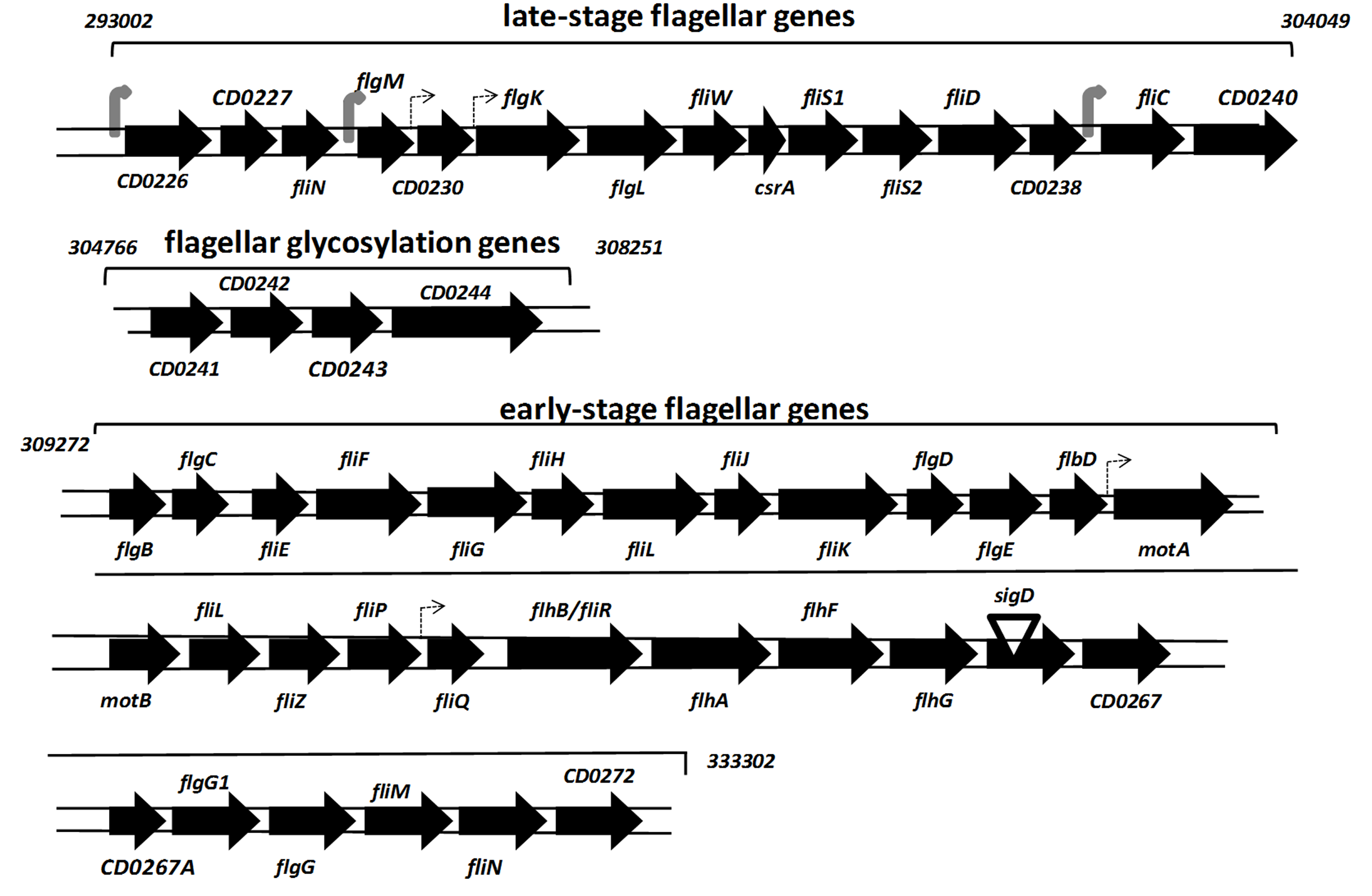

Supplement: Figure S1 — (adapted from Aubry et al [19]) : Flagellar locus from C. difficile 630, with location of the three SigD promoter sites identified by RACE-PCR (arrows above the flagellar locus). Dashed arrows indicate genes which posses a SigD consensus sequence and which significantly regulated by SigD. White triangle: mutagenesis of sigD gene using the Clostron system. (TIF) [file pone.0083748.s001.tif]

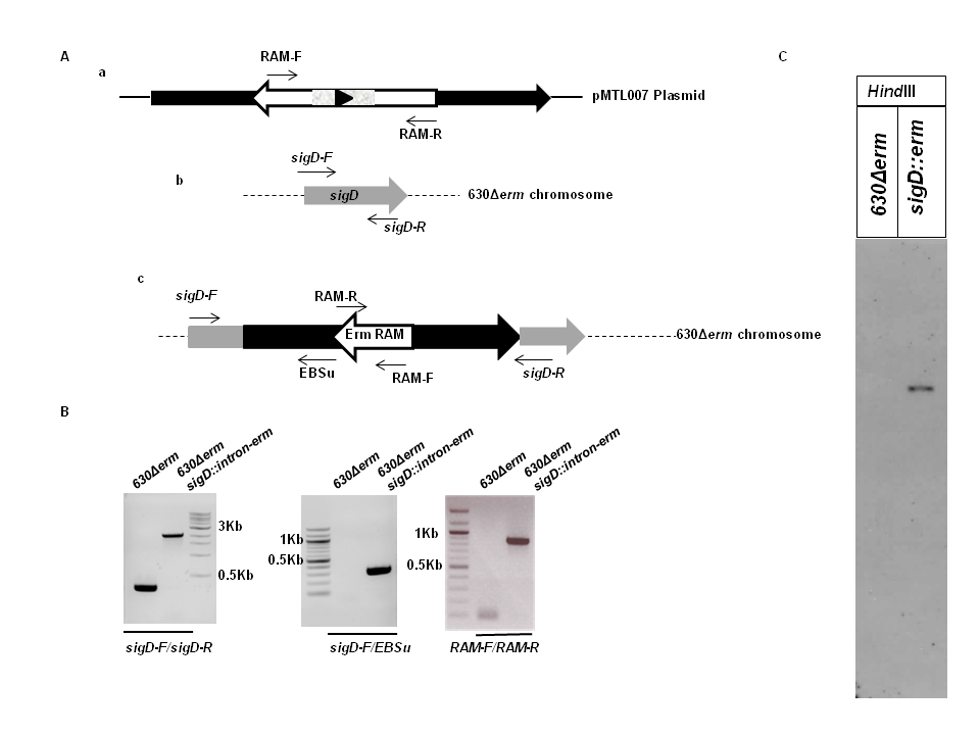

Supplement: Figure S2 — Inactivation of sigD gene. A: Schematic presentation of pMTL-based knocks-out plasmid. a: parental plasmid pMTL007. b: wild-type target gene. c: mutated target gene. Group II intron (black arrow), internal RAM conferring erythromycin resistance (white arrow) are represented.The locations of primers used for screening mutants are indicated. B: Confirmation of gene knockouts using PCR. Amplifications were performed on630Δerm and630Δerm sigD::intron-erm using: sigD target specific primers F and R (sigD-F and sigD-R), sigD-F and EBSu primers and ErmRAM-F and ErmRAM-Rprimers. C: Southern blot analysis of genomic DNA from C. difficile 630Δerm andC. difficile 630Δerm sigD::intron-erm with an intron probe. Chromosomal DNA (6µg in each reaction) was digested with HindIII. (TIF) [file pone.0083748.s002.tif]
