# Supplementary material for: Characterization of the SigD Regulon of C. difficile and Its Positive Control of Toxin Production through the Regulation of tcdR
Source: PLoS One. 2013 Dec 16;8(12):e83748. doi: 10.1371/journal.pone.0083748 (PMC3865298; doi:10.1371/journal.pone.0083748)
Supplement: Table S1 — Oligonucleotides used in this study. (DOCX) [file pone.0083748.s003.docx]

| **Name** | **Sequence (5’→3’)** | **Use** |
| --- | --- | --- |
| IBS-*sigD* | AAAAAAGCTTATAATTATCCTTAAGAAACCAAAGTGTGCGCCCAGATAGGGTG | *sigD*intron retargeting |
| EBS1d-*sigD* | CAGATTGTACAAATGTGGTGATAACAGATAAGTCCAAAGTCCTAACTTACCTTT | *sigD*intron retargeting |
| EBS2-*sigD* | TGAACGCAAGTTTCTAATTTCGATTTTTCTTCGATAGAGGAAAGTGTCT | *sigD*intron retargeting |
| EBS universal | CGAAATTAGAAACTTGCGTTCAGTAAAC | intron retargeting |
| Erm RAM-F | ACGCGTTATATTGATAAAAATAATAATAGTGGG | *Erm*-RAM |
| Erm RAM-R | ACGCGTGCGACTCATAGAATTATTTCCTCCCG | *Erm*-RAM |
| *sigD*-F | GGGGCAAAGTTTTCTAGTTAC | mutagenesis confirmation |
| *sigD*-R | GGAGCTTCCTCTTCTCTATCAC | mutagenesis confirmation |
| pMTLseq-F | GGGATCCTCTAGAGTCG | intron retargeted sequencing |
| pMTLseq-R | CAGATTCTCGGCATCGC | intron retargeted sequencing |
| pMTLcompF | GACCATGATTACGAATTCGAGC | Over-expression |
| pMTLcompR | GCTGCTGCATCTCTTCGC | Over-expression |
| P*2767*-F | AATAGTCTCGAGCGTTATAAAAACAGCAAAGAAG | Over-expression |
| P*2767*-R | TCATGAATTCCCCCTTAATTTTTTAAATTTATTTTTTC | Over-expression |
| *flgM*-F | GGAGGTGAATTCATGAATATTAAAAGTGTAAGTTC | Over-expression |
| *flgM*-R | TATGACCGATCGGTCTGTAATCATTACTTTATCCTCG | Over-expression |
| *sigD*comptetF | AAGAGAGCTCGTATATTTAAAAGCTAAGGAGGCG | Complementation |
| *sigD*comptetR | AATA GGATCCGTATCATCTTATCAATCA CCAT | Complementation |
| *CD0272*comptetR | TCTAGGATCCCTATTCTTCTTTTAAAATTTCTTTTACTTTTG | Complementation |
| F NF1323 | CTG GAC TTC ATG AAA AAC TAA AAA AAA TAT TG | Complementation |
| R NF794 | CACCGACGAGCAAGGCAAGACCG | Complementation |
| OS314 | CGGGATCCTTAAGCAATACTTAAAAGTAAACGC | *tcdR* overexpression |
| OS315 | CCCAAGCTTCTCTTTTATATATCCTCCTTTC | *tcdR* overexpression |
| *16S* RT-F | GAGGAAGGIGIGGAIGACGT | qRT-PCR |
| *16S* RT-R | AGICCCGIGAACGTATTCAC | qRT-PCR |
| *sigD* RT-F | AGAGATTCTATCCAAAGCAATTAG | qRT-PCR |
| *sigD* RT-R | TGTGAAACTCTTGATTCTGAAAC | qRT-PCR |
| *flgM* RT-F | GAGCGATGAAGCGGCTAG | qRT-PCR |
| *flgM* RT-R | TATCCTCGCATCTCCTCTATC | qRT-PCR |
| *fliC* RT-F | CTGATGATGCTGCTGGACTTG | qRT-PCR |
| *fliC* RT-R | TTCTTCTAACGAACCTTCTGCTG | qRT-PCR |
| *tcdA* RT-F | TCAATCCTGACACTGCTATCATCTC | qRT-PCR |
| *tcdA* RT-R | CCATTAAAGGCAATAGCGGTATCAG | qRT-PCR |
| *tcdB* RT-F | GGTAAATGTGAAATCTGGAGAATGG | qRT-PCR |
| *tcdB* RT-R | GTGTGGCTCTCTATATGTTATTGATG | qRT-PCR |
| *tcdR*RT-F | TAGCAAGAAATAACTCAGTAGATG | qRT-PCR |
| *tcdR* RT-R | TCTGTTTCTCCCTCTTCATAATG | qRT-PCR |
| *CD0240* RT-F | GGAAAGGCTAAGAAGTGTGA | qRT-PCR |
| *CD0240* RT-R | TCCCTAAATCAACTTGTGCT | qRT-PCR |
| *CD0244* RT-F | CCTAATGGCAAAGATGGTGTC | qRT-PCR |
| *CD0244* RT-R | CTCAAGTTCTAATGGATGTGGTC | qRT-PCR |
| *CD3527* RT-F | ATCAGGTGGACAACAACAAAGG | qRT-PCR |
| *CD3527* RT-R | GCAGATAAAGGTTCATCAAGAAGC | qRT-PCR |
| *CD0767* RT-F | CTTCTGTTGGTGAGTTAGTAAACC | qRT-PCR |
| *CD0767* RT-R | CCTACGCCAATAGGTGCTATTTC | qRT-PCR |
| *acd* RT-F | GTCTACACTTGCCCAAGGTCAAAC | qRT-PCR |
| *acd* RT-R | GCCATTGGAATACGCTGCTTCTG | qRT-PCR |
| *CD2767* RT-F | ATATGACACAGCAGTAGCAATAAG | qRT-PCR |
| *CD2767* RT-R | AGCAAGTGGTGTTGATGTTATAC | qRT-PCR |
| *CD1036* RT-F | CACAATACGACTCCAACAGAAC | qRT-PCR |
| *CD1036* RT-R | CATTCCTCCAGATGAATTTCCAG | qRT-PCR |
| *licC* RT-F | GGCTGGGGACACGATTAAAAC | qRT-PCR |
| *licC* RT-R | TTGGGTTTCTATCATTGGCTTCC | qRT-PCR |
| *sigH* RT-F | TAAGAGCAAGTGAAGGGGATAA | qRT-PCR |
| *sigH* RT-R | AAGGCCTATCATTCCTTCTTGT | qRT-PCR |
| sinR RT-F | AAGGCAGGTTTACATCCAACA | qRT-PCR |
| *sinR RT-R* | TTTGCAAATAACAATTCAAGTGG | qRT-PCR |
| *CD2215* RT-F | AAAAGACTTAAAGAAGAACGGAAAA | qRT-PCR |
| *CD2215* RT-R | TTGGATTCTTTTTACCACTTTCG | qRT-PCR |
| Spo0A RT-F | ATGTTGAGCTTTTAGGTGCAGT | qRT-PCR |
| Spo0A RT-R | CAACTTTTCCTCTACTCCATGC | qRT-PCR |
| Sp1-sigD | GCTTTGGATAGAATCTCTAAC | Race-PCR |
| Sp2-sigD | GCAATTTCATATGAAGTTGGCTC | Race-PCR |
| Sp3-sigD | CTTTGCCCCTTTTTCAGCATC | Race-PCR |
| Sp1-flgB | CCATTTGCAAAACTTATCAAAGC | Race-PCR |
| Sp2-flgB | CTACTCTTAAACCTTTAGAATTGTCTG | Race-PCR |
| Sp3-flgB | CTTAGATTAGTTGCATCTAATCCC | Race-PCR |
| Sp1-flgM | CCTCGCACTTCCTCTATC | Race-PCR |
| Sp2-flgM | CCGCTTCATCGCTCATATTTG | Race-PCR |
| Sp3-flgM | GTACCTTTTTTACATCCGATTTGTTC | Race-PCR |
| Sp1-CD3527 | CAATAGATATGTCTTTTAGCACC | Race-PCR |
| Sp2-CD3527 | CTCTAGTTAAATATTCTCACTTCC | Race-PCR |
| Sp3-CD3527 | CATGATATATAACACAAGTTCCC | Race-PCR |
| OS273 | AACAAATATCTTCCTCAAAAACAGA | *tcdR*Race-PCR |
| OS318 | TTTGACTATAATAGCATCGCTTTTT | *tcdR*Race-PCR |
| OS317 | GACTTTTGCATAAAATCATCCTC | *tcdR*Race-PCR |
| OS355 | TTTGAAAATTTTGACAACATTGGA | *tcdR*Race-PCR |
| *flgM*-surF | CATTCGGATCCATTAAAAGTGTAAGTTCAAATATTGTAG | Overexpression of *flgM* |
| *flgM*-surR | CATTCAAGCTTCTGTAATCATTACTTTATCCACGC | Overexpression of *flgM* |
| *sigD-*surF | CATTCGGATCCAGAGAAGAATTAATAAAAGAGAATATGC | Overexpression of *sigD* |
| *sigD-*surR | CATTCAAGCTTCATCTTATCAATCACCATCTATATAG | Overexpression of *sigD* |
| *tcdR up*F | CTTTAATAAAATGATTTGTTTTTACAATAC | Gel retardation |
| *tcdR up*R | GACTTTTGCATAAAATCATCCTC | Gel retardation |
| OBD522 | ATCTGTAGGAGAACCTATGGGAAC | Southern blot probe |
| OBD523 | CACGTAATAAATATCTGGACGTAAAA | Southern blot probe |
