# Supplementary material for: Characterization of the SigD Regulon of C. difficile and Its Positive Control of Toxin Production through the Regulation of tcdR
Source: PLoS One. 2013 Dec 16;8(12):e83748. doi: 10.1371/journal.pone.0083748 (PMC3865298; doi:10.1371/journal.pone.0083748)
Supplement: Table S2 — Genes positively or negatively controlled by SigD according to the expression ratio in transcriptomic analysis of sigD mutant/strain 630∆erm after 6h of growth. (DOCX) [file pone.0083748.s004.docx]

| **Gene ID** | **Name** | **Product** | **Expression**  **ratio**  ***sigD*mutant/630Δ*erm*** |  |  |  |
| --- | --- | --- | --- | --- | --- | --- |
| **Cell factor** |  |  |  |  |  |  |
| CD0392 |  | Putative radical SAM-family protein | 2.22 |  |  |  |
| CD2613 |  | Putative peptidase, M24 family | 0.46 |  |  |  |
| CD2841 |  | Putative amidohydrolase | 0.22 |  |  |  |
|  |  |  |  |  |  |  |
| **Cell wall** |  |  |  |  |  |  |
| CD3145 | cbpA | Surface exposed adhesin | 0.10 |  |  |  |
| CD0514 | cwpV | Cell surface protein | 0.46 |  |  |  |
| CD1036 | cwp17 | Putative N-acetylmuramoyl-L-alanine amidase, autolysin | 0.69 |  |  |  |
| CD2518 | cwp29 | Cell surface protein | 0.73 |  |  |  |
| CD2767 | cwp19 | Putative cell surface protein | 0.78 |  |  |  |
| CD2789 | cwp66 | Cell surface protein | 0.72 |  |  |  |
| CD1304 | acd | Mannosyl-glycoprotein endo-beta-N-acetylglucosamidase | 0.80 |  |  |  |
| CD1010 | nagA | N-acetylglucosamine-6-phosphate deacetylase (GlcNAc 6-P deacetylase) | 0.69 |  |  |  |
| CD1011 | nagB | Glucosamine-6-phosphate deaminase | 0.61 |  |  |  |
| CD0393 |  | Putative membrane protein | 2.24 |  |  |  |
| CD2520 |  | Putative conjugative transposon antibiotic resistance protein | 0.62 |  |  |  |
| CD2141 |  | Serine-type D-Ala-D-Ala carboxypeptidase | 1.57 |  |  |  |
| CD0211 | licC | CTP:phosphocholine cytidylyltransferase | 0.05 |  |  |  |
| CD0212 |  | Putative sulfatase | 0.05 |  |  |  |
| CD0241 |  | Phosphoserine phosphatase | 0.04 |  |  |  |
| CD0242 |  | Conserved hypothetical protein | 0.04 |  |  |  |
| CD0243 |  | Conserved hypothetical protein | 0.04 |  |  |  |
| CD0244 |  | Putative CDP-glycerol:Poly(glycerophosphate) glycerophosphotransferase | 0.04 |  |  |  |
|  |  |  |  |  |  |  |
| **Membrane transport** |  |  |  |  |  |  |
| CD1653 |  | Putative Probable D-methionine-binding lipoprotein | 0.49 |  |  |  |
| CD3525 |  | ABC-type transport system, iron-family extracellular solute-binding protein | 0.04 |  |  |  |
| CD3526 |  | ABC-type transport system, iron-family permease | 0.06 |  |  |  |
| CD3527 |  | ABC-type transport system, iron-family ATP-binding protein | 0.04 |  |  |  |
| CD3373 | mgtA | Magnesium-transporting ATPase, P-type 1 Tn916-like, CTn7-Orf7 | 0.53 |  |  |  |
| CD3374 |  | Putative conjugative transposon protein Tn916-like, CTn7-Orf8 | 0.55 |  |  |  |
| CD3374A |  | Putative conjugative transposon protein Tn916-like, CTn7-Orf9 | 0.46 |  |  |  |
| CD3375 | mgtC | Magnesium-transporting ATPase protein C Tn916-like, CTn7-Orf10 | 0.52 |  |  |  |
| CD3036 |  | Transporter, Major Facilitator Superfamily (MFS) | 0.33 |  |  |  |
| CD0205 |  | Transcription antiterminator, PTS operon regulator | 3.85 |  |  |  |
| CD0206 |  | PTS system, fructose-like IIA component | 3.77 |  |  |  |
| CD0207 |  | PTS system, fructose-like IIC component | 4.12 |  |  |  |
| CD0208 |  | PTS system, fructose-like IIB component | 3.75 |  |  |  |
| CD0209 |  | Putative tagatose 6-phosphate kinase | 4.16 |  |  |  |
| CD2325 |  | PTS system, fructose/mannitol family IIC component | 0.27 |  |  |  |
| CD2326 |  | PTS system, IIB component | 0.18 |  |  |  |
| CD2327 | gatA | PTS system, fructose/mannitol family IIA component | 0.32 |  |  |  |
| CD2555 |  | PTS system, fructose/mannitol-family IIB component | 0.63 |  |  |  |
| CD2556 |  | PTS system, fructose/mannitol-family IIAB component | 0.64 |  |  |  |
| CD3027 |  | PTS system, glucose-like IIA component | 0.44 |  |  |  |
| CD3028 |  | Putative phosphosugar isomerase | 0.43 |  |  |  |
| CD3029 | malY | Bifunctional protein: cystathionine beta-lyase / repressor | 0.44 |  |  |  |
| CD3030 |  | PTS system, glucose-like IIBC component | 0.46 |  |  |  |
| CD3031 |  | Transcription antiterminator, PTS operon regulator | 0.57 |  |  |  |
| CD0491 |  | PTS system, mannose/fructose/sorbose IIA component | 0.61 |  |  |  |
| CD0492 |  | PTS system, mannose/fructose/sorbose IIB component | 0.57 |  |  |  |
| CD0493 |  | PTS system, mannose/fructose/sorbose IIC component | 0.58 |  |  |  |
| CD0494 |  | PTS system, mannose/fructose/sorbose IID component | 0.62 |  |  |  |
| CD0762 | srlR | Transcription antiterminator, PTS operon regulator | 2.34 |  |  |  |
| CD0763 | srlM | Sorbitol operon activator protein (Glucitol) | 2.73 |  |  |  |
| CD0764 | srlA | PTS system, sorbitol-specific IIC component (Glucitol) | 2.67 |  |  |  |
| CD0765 | srlEA | PTS system, sorbitol-specific IIB N-terminal component (Glucitol) | 3.33 |  |  |  |
| CD0766 | srlEb | PTS system, sorbitol-specific IIB C-terminal component (Glucitol) | 3.44 |  |  |  |
| CD0767 | srlB | PTS system, sorbitol-specific IIA component (Glucitol) | 2.99 |  |  |  |
| CD0768 | srlD | Sorbitol 6-phosphate 2-dehydrogenase (Glucitol) | 3.04 |  |  |  |
|  |  |  |  |  |  |  |
| **Metabolism Amino Acid** |  |  |  |  |  |  |
| CD2531 |  | Putative membrane protein | 3.62 |  |  |  |
| CD2532 |  | Aminotransferase, alanine--glyoxylate transaminase | 3.62 |  |  |  |
| CD1657 | gcvTPA | Bi-functional glycine dehydrogenase/aminomethyl transferase protein | 2.39 |  |  |  |
| CD1658 | gcvPB | Glycine decarboxylase | 2.54 |  |  |  |
| CD0995 | serA | Putative D-3-phosphoglycerate dehydrogenase | 3.09 |  |  |  |
| CD0996 |  | Conserved hypothetical protein | 1.91 |  |  |  |
|  |  |  |  |  |  |  |
| **Metabolism Carbon** |  |  |  |  |  |  |
| CD2600 | cstA | Carbon starvation protein, CstA | 0.43 |  |  |  |
| CD3640 |  | Putative ribokinase family sugar kinase | 0.22 |  |  |  |
| CD3641 |  | Conserved hypothetical protein | 0.26 |  |  |  |
| CD2323 |  | Putative sugar-phosphate dehydrogenase | 0.27 |  |  |  |
| CD2324 |  | Putative sugar-phosphate dehydrogenase | 0.28 |  |  |  |
|  |  |  |  |  |  |  |
| **Metabolism Nucleic Acid** |  |  |  |  |  |  |
| CD3642 |  | Putative selenocysteine synthase | 0.29 |  |  |  |
| CD3643 |  | Dihydroorotase | 0.27 |  |  |  |
|  |  |  |  |  |  |  |
| **Mobile Element** |  |  |  |  |  |  |
| CD3371 |  | Putative conjugative transposon protein Tn916-like, CTn7-Orf3 | 0.48 |  |  |  |
| CD3372 |  | Putative conjugative transposon protein Tn916-like, CTn7-Orf4 | 0.60 |  |  |  |
|  |  |  |  |  |  |  |
| **motility** |  |  |  |  |  |  |
| CD0226 |  | Putative lytic transglycosylase | 0.08 |  |  |  |
| CD0227 |  | Conserved hypothetical protein | 0.09 |  |  |  |
| CD0228 | fliN | Flagellar motor switch protein FliN | 0.09 |  |  |  |
| CD0229 | flgM | Negative regulator of flagellin synthesis (Anti-sigma-d factor) | 0.02 |  |  |  |
| CD0230 |  | Putative flagellar biosynthesis protein | 0.05 |  |  |  |
| CD0231 | flgK | Flagellar hook-associated protein FlgK (or HAP1) | 0.03 |  |  |  |
| CD0232 | flgL | Flagellar hook-associated protein FlgL (or HAP3) | 0.03 |  |  |  |
| CD0233 | fliW | Flagellar assembly factor FliW | 0.03 |  |  |  |
| CD0234 | csrA | Carbon storage regulator homolog CsrA | 0.03 |  |  |  |
| CD0235 | fliS1 | Flagellar protein FliS1 | 0.02 |  |  |  |
| CD0236 | fliS2 | Flagellar protein FliS2 | 0.02 |  |  |  |
| CD0237 | fliD | Flagellar hook-associated protein 2 FliD (or HAP2) | 0.02 |  |  |  |
| CD0238 |  | Conserved hypothetical protein | 0.03 |  |  |  |
| CD0239 | fliC | Flagellin C | 0.02 |  |  |  |
| CD0240 |  | Glycosyltransferase | 0.06 |  |  |  |
| CD0241 |  | Phosphoserine phosphatase | 0.04 |  |  |  |
| CD0242 |  | Conserved hypothetical protein | 0.04 |  |  |  |
| CD0243 |  | Conserved hypothetical protein | 0.04 |  |  |  |
| CD0244 |  | Putative CDP-glycerol:Poly(glycerophosphate) glycerophosphotransferase | 0.04 |  |  |  |
| CD0245 | flgB | Flagellar basal-body rod protein FlgB | 0.61 |  |  |  |
| CD0246 | flgC | Flagellar basal-body rod protein FlgC | 0.57 |  |  |  |
| CD0247 | fliE | Flagellar hook-basal body complex protein FliE | 0.59 |  |  |  |
| CD0248 | fliF | Flagellar M-ring protein FliF | 0.58 |  |  |  |
| CD0249 | fliG | Flagellar motor switch protein FliG | 0.56 |  |  |  |
| CD0250 | fliH | Flagellar assembly protein FliH | 0.52 |  |  |  |
| CD0251 | fliI | ATP synthase subunit beta FliI | 0.60 |  |  |  |
| CD0252 | fliJ | Flagellar protein FliJ | 0.51 |  |  |  |
| CD0253 | fliK | Flagellar hook-length control protein FliK | 0.54 |  |  |  |
| CD0254 | flgD | Basal-body rod modification protein FlgD | 0.61 |  |  |  |
| CD0255 | flgE | Flagellar hook protein FlgE (Distal rod protein) | 0.63 |  |  |  |
| CD0255A | FlbD | Flagellar protein FlbD | 0.63 |  |  |  |
| CD0256 | motA | Flagellar motor rotation protein MotA | 0.56 |  |  |  |
| CD0257 | motB | Flagellar motor rotation protein MotB (Chemotaxis protein MotB) | 0.58 |  |  |  |
| CD0258 | fliL | Flagellar basal body-associated protein FliL | 0.56 |  |  |  |
| CD0259 | fliZ | Flagellar protein FliZ | 0.57 |  |  |  |
| CD0260 | fliP | Flagellar biosynthesis protein FliP | 0.56 |  |  |  |
| CD0261 | fliQ | Flagellar biosynthetic protein FliQ | 0.51 |  |  |  |
| CD0262 | flhB | Bifunctional flagellar biosynthesis protein FliR/FlhB | 0.59 |  |  |  |
| CD0263 | flhA | Flagellar biosynthesis protein FlhA | 0.61 |  |  |  |
| CD0264 | flhF | Flagellar biosynthesis regulator FlhF (Flagella-associated GTP-binding protein) | 0.61 |  |  |  |
| CD0265 | flhG | Flagellar number regulator FlhG | 0.58 |  |  |  |
| CD0266 | sigD | RNA polymerase sigma-28factor for flagellar operon | 0.13 |  |  |  |
| CD0267 |  | Putative flagellar protein | 0.14 |  |  |  |
| CD0268 | flgG1 | Flagellar hook-basal body complex protein FlgG1 | 0.14 |  |  |  |
| CD0269 | flgG | Flagellar basal body rod protein FlgG | 0.14 |  |  |  |
| CD0270 | fliM | Flagellar motor switch protein FliM | 0.12 |  |  |  |
| CD0271 | fliN | Flagellar motor switch phosphatase FliN | 0.13 |  |  |  |
| CD0272 |  | Conserved hypothetical protein | 0.16 |  |  |  |
| CD0533 | cheY | Chemotaxis protein CheY | 0.46 |  |  |  |
| CD0534 | cheC | Chemotaxis protein CheY-P phosphatase CheC | 0.48 |  |  |  |
| CD0535 | cheD | Probable chemoreceptor glutamine deamidase CheD | 0.52 |  |  |  |
| CD0536 | cheW | Purine-binding chemotaxis protein CheW | 0.50 |  |  |  |
| CD0537 |  | Putative CheY-like chemosensory protein | 0.65 |  |  |  |
| CD0538 |  | Putative methyl-accepting chemotaxis receptor, MCP family | 0.60 |  |  |  |
| CD0539 | cheA | Chemotaxis protein CheA | 0.60 |  |  |  |
| CD0540 | cheW | Chemotaxis protein CheW | 0.54 |  |  |  |
| CD0541 | cheR | Methyl-accepting chemotaxis proteins methyltransferase, MCP family | 0.62 |  |  |  |
|  |  |  |  |  |  |  |
| **Regulations** |  |  |  |  |  |  |
| CD2214 | SinR | Transcriptional regulator, HTH-type | 5.65 |  |  |  |
| CD2215 |  | Transcriptional regulator, HTH-type | 3.75 |  |  |  |
| CD2530 |  | Transcriptional regulator, AraC family | 2.72 |  |  |  |
| CD0618 |  | Transcriptional regulator, LytR family | 3.88 |  |  |  |
| CD0616 |  | Transcriptional regulator, MerR family | 2.88 |  |  |  |
| CD0617 |  | Putative membrane protein | 3.38 |  |  |  |
| CD0615 |  | Transcriptional regulator, TetR family | 0.74 |  |  |  |
| CD2668 |  | Transcription antiterminator, licT family | 0.45 |  |  |  |
|  |  |  |  |  |  |  |
| **Secretion** |  |  |  |  |  |  |
| CD2127 |  | Putative exported protein | 0.25 |  |  |  |
| CD3366 |  | Putative exported protein | 0.28 |  |  |  |
|  |  |  |  |  |  |  |
| **Sporulation** |  |  |  |  |  |  |
| CD1579 |  | Two-component sensor histidine kinase, sporulation-associated spo0A | 0.69 |  |  |  |
| CD0124 | spoIID | Stage II sporulation protein D | 1.55 |  |  |  |
| CD2442 | spoIV | Stage IV sporulation protein | 1.49 |  |  |  |
| CD1213 | spoIVB | Stage IV sporulation protein B, peptidase S55 family | 1.44 |  |  |  |
| **Stress** |  |  |  |  |  |  |
| CD0273 | htpG | Heat shock protein 90 (Heat shock protein HtpG)(High temperature protein G) | 0.45 |  |  |  |
| CD1690 | trxA | Thioredoxin | 2.42 |  |  |  |
| CD1691 | trxB | Putative thioredoxin-disulfide reductase | 1.74 |  |  |  |
|  |  |  |  |  |  |  |
| **Translation** |  |  |  |  |  |  |
| CD3661A | rpsR | 30S ribosomal protein S18 | 1.57 |  |  |  |
| CD3662 | ssb | Single-stranded DNA-binding protein (Helix-destabilizing protein) | 2.08 |  |  |  |
| CD2562A | rpmB | 50S ribosomal protein L28 | 2.29 |  |  |  |
| **Unknown** |  |  |  |  |  |  |
| CD1156 |  | Conserved hypothetical protein | 0.10 |  |  |  |
| CD2529 |  | Conserved hypothetical protein | 2.25 |  |  |  |
| CD2752 |  | Conserved hypothetical protein | 2.70 |  |  |  |
| CD3043 |  | Conserved hypothetical protein | 0.21 |  |  |  |
| CD3271 |  | Conserved hypothetical protein | 0.50 |  |  |  |
| CD3367 |  | Conserved hypothetical protein | 0.18 |  |  |  |
| CD0619 |  | Conserved hypothetical protein | 0.24 |  |  |  |
| CD0620 |  | Conserved hypothetical protein | 0.15 |  |  |  |
| CD0622 |  | Conserved hypothetical protein | 0.13 |  |  |  |
| **PaloC** |  |  |  |  |  |  |
| CD0659 | tcdR | Alternative RNA polymerase sigma factors | 0.54 |  |  |  |
| CD0663 | tcdA | Toxin A | 0.24 |  |  |  |
